# Overlaps and distinctions between attention deficit/hyperactivity disorder and autism spectrum disorder in young adulthood: Systematic review and guiding framework for EEG-imaging research

**DOI:** 10.1016/j.neubiorev.2018.10.009

**Published:** 2019-01

**Authors:** Alex Lau-Zhu, Anne Fritz, Gráinne McLoughlin

**Affiliations:** Social, Genetic and Developmental Psychiatry Centre, Institute of Psychiatry, Psychology and Neuroscience, King’s College London, London, United Kingdom

**Keywords:** EEG, ERP, ADHD, Autism, Young adulthood, Neurodevelopmental disorders

## Abstract

Attention deficit/hyperactivity disorders (ADHD) and autism spectrum disorders (ASD) frequently co-occur. However, we know little about the neural basis of the overlaps and distinctions between these disorders, particularly in young adulthood – a critical time window for brain plasticity across executive and socioemotional domains. Here, we systematically review 75 articles investigating ADHD and ASD in young adult samples (mean ages 16–26) using cognitive tasks, with neural activity concurrently measured via electroencephalography (EEG) – the most accessible neuroimaging technology. The majority of studies focused on event-related potentials (ERPs), with some beginning to capitalise on oscillatory approaches. Overlapping and specific profiles for ASD and ADHD were found mainly for four neurocognitive domains: attention processing, performance monitoring, face processing and sensory processing. No studies in this age group directly compared both disorders or considered dual diagnosis with both disorders. Moving forward, understanding of ADHD, ASD and their overlap in young adulthood would benefit from an increased focus on cross-disorder comparisons, using similar paradigms and in well-powered samples and longitudinal cohorts.

## Highlights

• The neural basis of ADHD-ASD overlap is understudied in young adulthood (ages 16–26).
• We systematically reviewed relevant EEG-imaging studies (with cognitive tasks) since 2000.
• Seventy-five articles were identified covering seven broad neurocognitive domains.
• Findings suggest neural overlaps and distinctions between ADHD and ASD.
• Not a single study compared both disorders directly or considered dual-diagnosis.

## 1 Introduction

Attention deficit/hyperactivity disorder (ADHD) and autism spectrum disorders (ASD) are neurodevelopmental conditions that frequently co-occur. There is currently an intensive search for the neural basis of these disorders. Electroencephalography, or EEG, has emerged as an ideal cognitive neuroscience tool in developmental psychopathology research, as it is relatively non-invasive, inexpensive and accessible compared to other neuroimaging technologies (McLoughlin et al., 2014a). To date, ADHD and ASD have been largely investigated in separate research fields and with a predominant focus on childhood. Little work has been done in the transitional phase into adulthood or young adulthood. This is despite this period representing a critical time window for neurodevelopment, marked by neurobiological development in the association cortices and the frontolimbic system implicated across executive and socioemotional processes (Taber-Thomas and Pérez-Edgar, 2014). Our aim is to systematically review EEG-imaging research in ADHD and ASD in young adult samples – we hope to provide a comprehensive and timely review of the literature to guide future investigations into the neurocognitive basis of these disorders, as well as of their overlap and distinction.

### 1.1 ADHD-ASD overlap

ADHD is characterised by severe deficits in attention, hyperactivity and impulsivity, whereas ASD is associated with impaired communication and social interaction skills, in addition to repetitive and restricted behaviour and interests (American Psychiatric Association or APA, 2013). These two disorders frequently co-occur (Russell et al., 2014), with ADHD presenting in 30–80% of individuals with ASD, and ASD presenting in 20–50% of individuals with ADHD (van der Meer et al., 2012). Below-threshold cross-disorder symptoms are also common, that is, having symptoms of the other disorder despite not having the diagnosis (Ronald et al., 2014).

The phenotypic overlap between ADHD and ASD appears to be explained by aetiological overlap, at least partly, in terms of shared genetic influences between traits of both disorders. For example, individuals with ADHD and their siblings display more ASD symptoms than non-sibling controls, suggesting shared familiality (Mulligan et al., 2009). Twin studies further support shared genetic influences between traits of ADHD and ASD, increasing from 27% at age 2 (Ronald et al., 2010), to around 50% at age 8 (Ronald et al., 2008) and 72% at ages 18–33 (Reiersen et al., 2008). Research on molecular genetics have identified candidate genomic regions and genetic pathways implicated in this shared heritability, yet findings remain inconsistent, potentially because of insufficient statistical power in those studies (see Rommelse et al., 2010 for a comprehensive review).

Shared etiology between ADHD and ASD also points towards shared neurocognitive pathways. However, these disorders have, to date, been investigated in divergent fields. One reason is that dual diagnosis of ADHD and ASD was not formally permitted until the revision of Diagnostic and Statistical Manual of Mental Disorders in 2013 or DSM–5 (APA, 2013). However, given, that awareness of the relevance of ADHD and ASD to each other has dramatically increased in the last decade, demand has emerged to consolidate understanding of the overlap and distinction in neurocognitive processes between ADHD and ASD.

### 1.2 ADHD and ASD in young adulthood

The ‘in-between’ phase between adolescence and fully-fledged adulthood is known as *young adulthood* or *emerging adulthood* (Taber-Thomas and Pérez-Edgar, 2014). Young adults typically navigate the world to achieve independence and in doing so, they encounter an increase in social and executive demands alongside a reduction in parental scaffolding (Arnett, 2007; Stroud et al., 2015). Emerging adulthood represents a critical time period for increased brain plasticity (Arnett, 2007; Giedd, 2008; Rubia et al., 2000; Sowell et al., 2003; Taber-Thomas and Pérez-Edgar, 2014) and also for heightened risk of mental health difficulties (Kessler and Berglund, 2005; Patel et al., 2007).

ADHD and ASD are typically described as childhood disorders; yet, the transition from childhood to adulthood can highlight particular challenges in those with these diagnoses (Davidson, 2008; El Achkar and Spence, 2015). Compared to typically-developing individuals, adults with ADHD and/or ASD are at higher risk of experiencing a range of behavioural and cognitive problems, such as mood disorders, sleep problems and unfavourable psychosocial outcomes, including poorer academic performance and lower employment levels (Davidson, 2008; Levy and Perry, 2011). Having concurrent symptoms of both ASD and ADHD is associated with even poorer cognitive, emotional and functional outcomes than a single diagnosis (Anckarsäter et al., 2006; Murray, 2010; van der Meer et al., 2012).

Given the fact that dual-diagnosis of ADHD and ASD was not permitted until recently, ADHD is likely to have been underdiagnosed in adults who currently have ASD and vice versa (Ginsberg et al., 2014), which could potentially affect the development and availability of relevant interventions. We know little about the outcomes in young adults with single or dual-diagnosis, or in those who had received a diagnosis of either of these disorders initially in childhood. For example, despite the drop in diagnosis of ADHD in adulthood, significant and impairing symptoms frequently remain (Asherson, 2016). Nevertheless, there is relative paucity of services available to adults with these disorders compared to dedicated child/adolescent clinical services (Murphy et al., 2016; Nutt et al., 2007).

Understanding the neurocognitive basis of the developmental period in early adulthood is critical to expand our knowledge of the aetiology and course of ASD and ASHD, including their co-occurrence and potentially their associated negative outcomes.

### 1.3 EEG-imaging in ADHD and ASD research

EEG represents the most non-invasive, inexpensive and portable brain imaging tool available today (McLoughlin et al., 2014a). Originating in the 1920’s, EEG has had a renaissance since early 2000’s, emerging as a popular and accessible technology for mapping out neurocognitive mechanisms underlying neurodevelopmental disorders. EEG-based measures may shed light on the neurocognitive pathways that mediate shared genetic influences between different disorders, including those between ADHD and ASD.

EEG provides superlative temporal resolution of brain activity in the range of milliseconds, the timescale in which many perceptual and attentional processes are thought to operate (Kappenman and Luck, 2012; Makeig et al., 2004; Rugg and Coles, 1996). Its spatial resolution is traditionally regarded as low, but is continuing to improve at a cortical level due to advances in computational neuroscience and signal processing (Delorme et al., 2012; Jung et al., 2001).

EEG has traditionally been analysed in two ways. In the time domain, event-related potential (ERP) refers to transient time-locked EEG activity typically averaged across trials. Broadly speaking, early ERP components (100–200 ms) are thought to index stimulus-driven sensory processing, whereas later components are thought to index cognitive processing (Banaschewski and Brandeis, 2007; Kappenman and Luck, 2012). In the frequency domain, quantitative EEG (qEEG) refers to rhythmic cycles (per second, i.e., Hz), also known as brain oscillations, in frequency bands typically denoted as delta (1–4 Hz), theta (4–8 Hz), alpha (8–12 Hz), beta (13–30 Hz) and gamma (30–70 Hz). These are thought to signal various states such as wakefulness, arousal and cognition (Buzsáki, 2006; Klimesch, 1999). Innovative techniques combining both time and frequency-based information are rapidly expanding (Makeig et al., 2004) and recently harnessed in psychiatric research (McLoughlin et al., 2014b; Milne et al., 2009).

EEG-imaging has been extensively used to investigate neural mechanisms underlying ADHD and ASD, but to date most of this work has been in children and adolescents. Broadly speaking, ERP research has highlighted atypical ADHD-linked cognitive profiles in inhibitory control and performance monitoring (Barry et al., 2003b; Johnstone et al., 2013; Tye et al., 2011) and atypical ASD-linked cognitive profiles in socioemotional processing and executive function (Jeste and Nelson, 2009). QEEG research has demonstrated atypical ADHD-linked profiles mostly in relation to the theta and beta bands (Barry et al., 2003a; Tye et al., 2011) and atypical ASD-linked profiles mostly in relation to the alpha, beta, and gamma bands (Billeci et al., 2013). There is currently an increased interest in the neural basis of the co-occurrence of ADHD and ASD (Johnson et al., 2015; Rommelse et al., 2011), with cross-disorder comparisons using EEG-imaging rapidly emerging (Tye et al., 2014a, 2014b, 2013). Despite the importance of investigating these disorders in the key transitional stage of young adulthood, such relevant research is lacking in comparison to childhood studies.

### 1.4 Aim and scope

Our main goal is to systematically review studies using EEG-imaging in young adults with ADHD and/or ASD, focusing on both ERPs and EEG oscillatory measures. We aim to synthesize the numerous neurocognitive domains considered for each disorder independently, as well as when both disorders were directly compared and/or dual-diagnosis was considered. We include research focusing on diagnostic categories and traits to understand the mechanisms underlying these disorders more broadly. Traits and diagnosis appear to share common genetic origins, with diagnoses aetiologically represented as extreme ends of a continuum of behavioural traits (Colvert et al., 2015; Martin et al., 2014). The current state of this research could then be used to provide a guiding framework, as informed by EEG methods, for critical next steps in research investigating the neural basis of the overlap, and distinction, between ADHD and ASD during young adulthood.

Our review is restricted to EEG recorded during cognitive tasks, which allows for the interpretation of brain activity in the context of putative cognitive operations being carried out. EEG during cognitive tasks is more reliable (McEvoy et al., 2000) while the value of resting state research has been questioned (Morcom and Fletcher, 2007). Readers can refer to other existing reviews in ADHD and/or ASD for the use of EEG in resting state paradigms (Loo and Barkley, 2005; Wang et al., 2013) and neurofeedback interventions (Gevensleben et al., 2014; Holtmann et al., 2011), or for the use of EEG to investigate sleep states (Lustenberger and Huber, 2012) and epilepsy/seizures (Askamp and van Putten, 2014; Noachtar and Rémi, 2009), which are beyond the scope of our review. Meta-analyses are not conducted due to the heterogeneity in EEG and ERP features as well as cognitive paradigms employed.

Other imaging modalities are also beyond our current scope, but readers can refer to relevant reviews on the use of methods such as functional magnetic resonance imaging (fMRI) (Cortese et al., 2012; Paloyelis et al., 2007) and positron emission tomography (PET) (Krause, 2008) in ADHD, and of fMRI (Philip et al., 2012), PET (Zürcher et al., 2015) and magnetoencephalography (MEG) (Kikuchi et al., 2016) in ASD.

## 2 Method

### 2.1 Search strategy

Initial literature search and selection of studies were informed by guides from PRISMA (Preferred Reporting Items for Systematic Reviews and Meta-Analyses) (Welch et al., 2012). Searches were performed on 20/04/2017 using PubMed and Web of Science databases. Search terms were: (ADHD OR attention deficit hyperactivity disorder OR autism spectrum disorder OR autism OR ASD) AND (EEG OR ERP OR electrophysiology OR neurophysiology) NOT (epilepsy OR epileptic OR seizure) NOT (mouse OR mice OR rodent). The search was limited from the year 2000 and onward, the time around which we observed a resurgence of EEG research, particularly in the field of psychopathology (McLoughlin et al., 2014a). Titles and abstracts were used for initial screening. These filters were applied: written in English only and no case reports. We subsequently supplemented this initial search with additional searches using terms employed for earlier subgroup classifications of ASD in DSM-IV (Asperger, childhood disintegrative disorder, and pervasive developmental disorder not otherwise specified or PDD-NOS), but no additional eligible studies relevant to ASD were identified.

### 2.2 Inclusion and exclusion criteria

Inclusion criteria were 1) empirical research published in peer-reviewed journals; 2) involving human participants; 3) EEG was elicited within a cognitive task; 4) the study examined case-control differences between individuals with ADHD/ASD as defined by either research/clinical diagnosis or trait measures, and/or examined traits of ADHD/ASD-traits as continuous variables; 5) mean age was between ages 16–26 (or fell within the age range reported in a study and the study focused on adults). Exclusion criteria were if the study 1) did not use EEG-imaging, 2) focused on resting states, neurofeedback, sleep states, and/or epilepsy/seizures and/or 3) had a sample size of fewer than 12 participants with ADHD/ASD.

### 2.3 Structure of the findings

Out of 3738 articles identified in the initial search (PubMed = 1876; Web of Science = 1862), 3608 were discarded after screening and 75 articles were included in the current review (35 for ADHD and 40 for ASD). To organise this vast literature, the relevant studies are grouped according to the primary neurocognitive domain investigated, broadly defined as: 1) attention processing, 2) inhibitory control, 3) performance monitoring, 4) face processing, 5) imitation and empathy, 6) sensory processing, and 7) memory and language.

Each of the neurocognitive domains is subsequently divided according to the focus on ADHD or ASD. ERP studies are described first, followed by those including data relevant to brain oscillations (i.e., qEEG and/or time-frequency analyses). We did not find any research in the relevant age period directly comparing both disorders or considering dual-diagnosis. This points towards a critical gap in the literature for ADHD and ASD research in young adulthood – we return to this issue in our discussion, where we highlight the candidate neurocognitive processes that are likely to inform research investigating the ADHD-ASD overlap and/or distinction in young adulthood.

Details on the most prominent ERP and EEG oscillatory measures (qEEG and time-frequency approaches) discussed in this review, and their putative functional significance and/or definition, can found in Table 1A, Table 1B. We are aware that the interpretations of various ERPs and oscillatory features may be topics of ongoing debate, and thus Table 1A, Table 1B are included mainly to provide an initial guide for readers less familiar with the vast ERP/EEG literature.

**Table 1A:** Overview of main event-related potentials (ERP) from studies in the current review.

Neurocognitive domain	Prominent components	Description	Proposed functional significance	Relevant reference	Example of studies in the current review
Sensory processing	P50	Positive-going wave over fronto-temporal electrodes peaking at 40-75 ms poststimulus	Attenuation of this component indexes sensory gating (the ability to filter out redundant sensory information)	(Potter et al., 2006)	(Magnée et al., 2009)
	N1 (N100)	Negative-going wave over frontocentral electrodes peaking at 80-120 ms poststimulus	Sensory processing of unexpected (auditory) stimulus	(Rosburg et al., 2008)	(Gonen-Yaacovi et al., 2016; Sable et al., 2013)
	P1 (P100)	Positive-going wave over lateral occipital electrodes peaking at 80-130 ms poststimulus	Sensory processing of stimulus in the contralateral visual field	(Hillyard and Anllo-Vento, 1998)	(Gonen-Yaacovi et al., 2016; Sable et al., 2013)
	N170	Negative-going wave over posterior electrodes peaking around 130-200 ms poststimulus (face vs non-face)	Structural encoding of faces	(Feuerriegel et al., 2015)	(O’Connor et al., 2005; Stavropoulos et al., 2016)
	Early posterior negativity (EPN)	Negative-going wave peaking at 150–300 ms poststimulus	Selective visual attention toward emotional stimuli	(Citron, 2012)	(Faja et al., 2016)
	Mismatch negativity (MMN)	Negative-going wave peaking at 150-250 ms poststimulus	Detection of infrequent and odd deviant stimulus in a repetitive sequence of auditory or visual stimuli	(Garrido et al., 2009)	(Fan and Cheng, 2014)

Stimulus evaluation	N2	Negative-going wave peaking at 200-350 ms poststimulus	Detection of mismatch and/or inhibition of competing response	(Folstein and Van Petten, 2008)	(Fisher et al., 2011; Groom et al., 2008)
	N250	Negative-going wave over inferior temporal electrodes around 250 ms poststimulus when comparing familiar vs unfamiliar faces	Storage of face representation in long-term memory	(Eimer and Holmes, 2007)	(Webb et al., 2010)
	P2	Positive-going wave peaking at 100-250 ms poststimulus	Sensitivity to various stimulus features	(Crowley and Colrain, 2004)	(Chien et al., 2017; Megnin et al., 2012)
	P3a (novelty P3)	Positive-going wave over frontocentral electrodes peaking at 250-280 ms poststimulus	Novelty processing and involuntary orienting of attention	(Polich, 2007)	(Sokhadze et al., 2009b)
	P3b (classic P3)	Positive-going wave over parietal electrodes peaking at 250-500 ms poststimulus	Attentional engagement and stimulus evaluation/decision-making	(Polich, 2007)	(Iacono et al., 2002; Rommel et al., 2017)
	N3 (slow wave or late posterior negativity (LPN))	Negative-going wave peaking at 500-650 ms poststimulus over posterior electrodes	Enhanced attention to stimulus, particularly the nonautomatic, controlled part of the stimulus processing	(Johansson and Mecklinger, 2003)	(Raz and Dan, 2015b)
	N400	Negative-going wave over centropariental electrodes peaking at 250-500 ms poststimulus	Processing of semantic information	(Kutas and Federmeier, 2011)	(Gold et al., 2010; Kulakova and Nieuwland, 2016)
Response preparation	CNV	Negative-going wave rising around 260-470 ms after a warning stimulus	Response and motor preparation to upcoming stimulus	(Mento, 2013)	(Boecker et al., 2014; Cheung et al., 2016)
	Lateralised readiness potential (LRP)	Negative-going wave over motor cortices contralateral to the responding hands	Motor preparation before action execution	(Smulders and Miller, 2012)	(Gorman Bozorgpour et al., 2013)
	Late positive potential (LPP)	Positive-going slow wave arising around 600 ms poststimulus	Salience of emotional stimuli	(Olofsson et al., 2008)	(Althaus et al., 2015; Fan et al., 2014)

Error detection	Error-related negativity (ERN)	Negative-going wave rising 50–100 ms following erroneous response execution over frontocentral electrodes	Unconscious error processing	(Nieuwenhuis et al., 2001)	(Michelini et al., 2016; O’Connell et al., 2009)
	Error-related positivity (PE)	Positive-going wave over centro-parietal electrodes peaking around 200-500 ms post-error, after the occurrence of ERN	Conscious error processing	(Wessel, 2012)	(Michelini et al., 2016; O’Connell et al., 2009)

*Note*. ERP refers to transient time-locked EEG activity typically averaged across trials. Relevant quantitative measures include amplitude (in voltage, interpreted as engagement of a particular cognitive process) and latency (in ms, interpreted as timing of a particular cognitive process) (Kappenman and Luck, 2012).

**Table 1B:** Overview of main EEG oscillatory approaches from studies in the current review.

Analytical approach	Prominent components	Description	Proposed functional significance	Relevant references	Example of studies in the current review
Quantitative EEG (qEEG)	Very-low frequency (VLF)	EEG oscillations at 0.02-0.2 Hz	Default-mode network	(Sonuga-Barke and Castellanos, 2007)	(Broyd et al., 2011; Hsu et al., 2013)
	Delta	EEG oscillations at 1-4 Hz	Attention and inhibition	(Güntekin and Başar, 2016)	(Gilmore et al., 2011)
	Theta	EEG oscillations at 4-8 Hz	Cognitive control, learning and memory	(Sauseng et al., 2010)	(Groom et al., 2010; Sohn et al., 2010)
	Alpha	EEG oscillations at 8-12 Hz, typically over the occipital cortex	Alertness, attention and inhibition	(Klimesch et al., 2007)	(Groom et al., 2010; Sohn et al., 2010)
	Mu rhythms	EEG oscillations at 8-13 Hz and recorded over the sensorimotor cortex	Action execution and observation of others' actions	(Fox et al., 2016)	(Bernier et al., 2007; Thillay et al., 2016)
	Beta	EEG oscillations at 13-30 Hz	Sensorimotor processing and sensory gating	(Pogosyan et al., 2009)	(Nowicka et al., 2016)
	Gamma	EEG oscillations at 30-70 Hz	Sensory binding	(Buzsáki and Wang, 2012)	(Dickinson et al., 2015; Snijders et al., 2013)
Time-frequency analyses	Evoked power	Event-related changes in EEG power at a given frequency band, both time-locked and phase-locked to the event	Dynamic changes in power of a given frequency band over time	(Makeig et al., 2004)	(Gilmore et al., 2011)
	Induced power	Event-related changes in EEG power at a given frequency band, phase-locked but not time-locked to the event; also known as event-related synchronization (ERS, increase in power) or event-related desynchronization (ERD, decrease in power)	Dynamic changes in power of a given frequency band over time	(Makeig et al., 2004)	(Dickinson et al., 2015; Liu et al., 2016)
	Event-related phase-locking OR inter-trial coherence (ITC)	Phase similarity across trials in relation to the timing of the event at a given frequency band and within a given electrode	Consistency of timing of the event-related oscillations across trials (e.g., neural variability)	(Makeig et al., 2004)	(Gonen-Yaacovi et al., 2016)
	Coherence	Synchronicity of the EEG signals in the same frequency band between two separate electrodes, analogous to the Pearson product-moment-correlation coefficient	Brain's regional connectivity and interregional interaction	(Srinivasan et al., 2007)	(Nowicka et al., 2016)
	Cross-frequency coupling	Synchronicity of the EEG signal in two different frequency bands	Brain's regional connectivity and interregional interaction	(Canolty and Knight, 2010)	(Jochaut et al., 2015)

*Note.* Brain oscillations refer to rhythmic cycles per second (Hz) of brain activity (i.e., frequency). Relevant quantitative measures include power (refers to the square of amplitude and is interpreted as the dominance of a particular oscillation) and phase (refers to at which time point an oscillation is within its cycle). Time-frequency approaches refer to the combination of frequency approaches and time approaches (e.g., event-related) (Buzsáki, 2006).

Primary findings are summarised in terms of either significant differences between ADHD/ASD and non-ADHD/ASD control participants specific to the study, unless otherwise specified (e.g., additional clinical groups), and/or significant associations between ADHD/ASD traits and ERP/EEG measures. These are then interpreted, whenever possible, based on functional significance for neurocognitive processing. More details on the sample composition of each study can be found in subsequent tables, which also provide details on age (mean and range) and sample size of the ADHD/ASD group, information about the comparison group(s), cognitive paradigms employed, and ERP/EEG measures investigated. When multiple cognitive domains are relevant, studies are presented only under the primary category in the subsequent tables.

## 3 Results

### 3.1 Attention processing

Sixteen articles investigated attention processing, including indices of attentional orienting, subsequent attentional allocation and sustained attention. All except two studies considered clinical or research diagnosis. Thirteen of the studies focused on ADHD and three focused on ASD. The most widely used cognitive paradigms to study attentional processing in ADHD were visual and auditory oddball paradigms, continuous performance task (CPT) and, to a more limited extent, a four-choice reaction time task called the fast task (see Table 2A).

**Table 2A:** Studies on ADHD or ASD using EEG-imaging to investigate *attentional processing*.

Study	Clinical group	Diagnosis or trait	Age: Mean (SD)	Age range (years)	Sample size(s)	Comparison group(s) (sample size)	Cognitive paradigm	Analytical method	Features analysed
Iacono et al., 2002^a^	ADHD	D	17.5 (0.4)	16 to 18	45	CD (185), ODD (87), ND (69), AAD (95), DAD (36)	Visual oddball	ERP	P3b
Groom et al., 2008	ADHD	D	15.7 (1.5)	14 to 21	27	E-SCZ (30), NPS (36), controls (36)	Auditory oddball & go/no-go	ERP	P3b, N2
Barry et al., 2009	ADHD	D	21.9 (1.8)	18 to 26	18	Non-ADHD (18)	Visual oddball	ERP	P1, N1, P2, N2, P3b
Sawaki and Katayama, 2006	ADHD	T	23.3 (2.8)	20 to 32	23	–	Visual oddball	ERP	P3b
Godefroid and Wiersema, 2016	ADHD	D	24.0 (5.3)	–	25	Non-ADHD (23)	Visual oddball	ERP	P2, N2, P3a, P3b
Rommel et al., 2017	ADHD	D	18.5 (3.0)	13 to 26	69	PTB (186), controls (135)	Cued CPT	ERP	P3b, N2, CNV
Cheung et al., 2016	ADHD	D	ADHD-P: 18.3 (3.0); ADHD-R: 18.9 (3.1)	–	ADHD-P: 87; ADHD-R: 23	Non-ADHD (168)	Cued CPT	ERP/oscillations	CNV, P3b, delta, theta, alpha, beta
Du Rietz et al., 2016	ADHD	D	ADHD-P: 18.5 (2.9); ADHD-R: 18.3 (3.2)	11 to 25	ADHD-P: 48; ADHD-R: 60	Non-ADHD (167)	Cued CPT	ERP/oscillations	CNV, P3b, delta, theta, alpha, beta
Cheung et al., 2017	ADHD	D	18.3 (3.0)	–	93	Non-ADHD (174)	Fast task	ERP	P3b, CNV
James et al., 2017	ADHD	D	ADHD-P: 18.1 (2.9); ADHD-R: 19.1 (2.7)	–	ADHD-P: 73; ADHD-R: 18	Non-ADHD (144)	Fast task	ERP	P3b, CNV
Gilmore et al., 2011^a^	ADHD	D	17.5 (0.4)	16 to 18	45	CD (185), ODD (87), ND (69), AAD (95), DAD (36)	Visual oddball	oscillations	TF-PCA of P3b
Broyd et al., 2011	ADHD	D	20.6 (1.6)	–	20	Non-ADHD (20)	Eriksen flanker task	oscillations	VLF
Hsu et al., 2013	ADHD	D	22.8 (3.8)	18 to 43	16	Non-ADHD (16).	Two-choice reaction time task	oscillations	VLF
Karhson and Golob, 2016	ASD	D	22.5 (4.1)	–	12	Non-ASD (13)	Auditory oddball	ERP	P50, N1, N2, P3b
Sokhadze et al., 2009a	ASD	D	17. 2 (4.6)	12 to 27	13	Non-ASD (13)	Visual oddball	ERP/oscillations	P3a, P3b, gamma
Milne et al., 2013	ASD	T	–	18 to 37	36	–	Target detection task	ERP/oscillations	P1, P3b, gamma

*Note*. EEG = electroencephalography; ADHD = attention deficit/hyperactivity disorder; ASD = autism spectrum disorder; D = diagnosis; T = trait; ERP = event related potentials; CD = conduct disorder; ODD = oppositional defiant disorder; ND = nicotine dependence; ADD = alcohol abuse or dependence; DAD = illicit drug abuse or dependence; E-SCZ = Early-onset schizophrenia; NPS = non-psychotic siblings of schizophrenia patients; PTB = pre-term born; ADHD-P = ADHD persisters; ADHD-R = ADHD remitters; CPT = continuous performance task; CNV = contingent negative variation; TF-PCA = time frequency principal component analysis; VLF = very-low frequency EEG; ^a^ These studies have the same sample.

#### 3.1.1 ADHD

##### 3.1.1.1 ERP

Several ERP studies have examined selective attention using oddball paradigms. In such studies, participants are typically presented with a sequence of standard auditory/visual stimuli intercepted with additional targets appearing less frequently. The Minnesota Twin Family Study (Iacono et al., 2002) examined the target-P3b in a visual oddball paradigm and found a reduced P3b amplitude for ADHD at age 17. The P3b ERP is believed to index reduced attentional allocation (Table 1A), and thus this study is in line with similar pattern of findings from studies with children and adolescents with ADHD (Barry et al., 2003b; Johnstone et al., 2013). However, such findings on target-P3b may not be ADHD-specific: the same pattern of reduced P3b amplitude in an oddball paradigm was found for other clinical groups, including oppositional defiant disorder, conduct disorder, antisocial personality disorder and those with paternal risk of these disorders (Iacono et al., 2002). Other studies failed to find an association between ADHD and target-P3b with oddball paradigms using auditory stimuli in ADHD at age 16 (Groom et al., 2008), or using combining visual standard stimuli and auditory targets in ADHD at age 22 (Barry et al., 2009). Thus, the modality of the stimuli, at least in oddball paradigms, may be an important consideration for EEG-imaging markers of ADHD.

Two additional oddball studies in adults aged 23–24 years included two types of nontargets: constant nontargets, for which the same nontarget stimulus was repeatedly presented, and novel nontargets, for which a completely novel stimulus was presented each time (i.e., never repeated). One study found that higher number of ADHD symptoms in a nonclinical sample were associated with more pronounced P3b amplitude for nontargets relative to targets, but only when constant nontargets rather than novel nontargets were considered (Sawaki and Katayama, 2006). This suggests that ADHD is associated with atypical attentional allocation to task-irrelevant stimuli more generally, regardless of their novelty. In contrast, another study using a clinical sample instead found that ADHD was associated with reduced P3b amplitude to novel nontargets, suggesting that attentional resources are inefficiently allocated to novel stimuli specifically (Godefroid and Wiersema, 2016). Such discrepant patterns of findings suggest that ERP markers of ADHD, at least in the context of oddball studies, may vary depending on whether ADHD traits or diagnosis are considered, and thus both should be assess simultaneously in future studies.

The cued CPT (i.e., CPT-OX) is a variant of the CPT task that is used widely in ADHD research to index aspects of attentional orienting, response preparation and response inhibition (e.g., McLoughlin et al., 2010). In this task, participants monitor a sequence of letters and are asked to respond whenever the target ‘X’ follows the cue letter ‘O’ known as ‘go’-trials. ‘No-go’ or inhibitory trials, however, are those when the cue letter ‘O’ is not followed by the target letter ‘X’. One study using the cued CPT task found overlapping and distinct ERP features between ADHD and preterm-born individuals aged between 13–26 years (Rommel et al., 2017). Both groups showed reduced amplitude of the contingent negative variation (CNV), a late component with a low frequency that is thought to index reduced response preparation for a subsequent response (Table 1A). Both groups also showed attenuated P3b amplitude time-locked to no-go trials, or the no-go-P3, which indicates reduced response inhibition processing. However, only those with ADHD showed reduced cue-P3b, which may index impaired attentional orienting to cue, and only preterm-born individuals showed reduced go-P3b, which in turn may index reduced cognitive control including resource allocation. Thus, atypical profiles of cue-P3b may be a more specific marker of ADHD as a disorder, rather than a marker of ADHD risk, as pre-term birth is a risk factor for ADHD (Bhutta et al., 2002).

The cued CPT has also been used to tease apart potential markers of persistence or remission of ADHD diagnosis or parental reports of ADHD symptoms at ages 18–19 with an earlier diagnosis of childhood ADHD (Cheung et al., 2016). ADHD persistence (having ADHD symptoms in both childhood and young adulthood) but not remittance (having symptoms in childhood only) was linked to reduced CNV amplitude and reduced cue-P3b amplitude. Further, ADHD persistence was also associated with reduced no-go-P3b amplitude, an index of response inhibition, whereas ADHD remittance was intermediate between ADHD persisters and non-ADHD controls in this ERP measure. Importantly, it was noted that the ERP differences between persisters and remitters disappeared when ADHD status was classified based on self-reported symptoms (Du Rietz et al., 2016), which may point towards potential issues with reliability of self-reports versus parent/informant reports in relation to EEG biomarkers of the disorders, an issue we return to in the discussion (Section 4).

Two ERP studies have used the fast task (Kuntsi et al., 2005). The task follows a standard warned four-choice reaction time task: on each trial, four empty circles are presented and then one of them is coloured in; participants are asked to press a key that corresponds on a keyboard to the location of the coloured circle on the screen. The baseline condition shows four circles that appear for 8 s before becoming filled with yellow. In the fast-incentive condition, the circles appear for 1 s before becoming coloured in with yellow; in this condition, smiley faces are awarded at the end of the trial if RTs are faster than in the baseline condition.

In one study, ADHD at age 18 was associated with reduced target-P3b amplitude in the baseline condition of the fast task, which in turn was also linked to higher RT variability (RTV), both of which suggest inefficient and inconsistent attentional allocation to targets (Cheung et al., 2017). In addition, the fast-incentive condition (with rewards) of the fast task, compared to the baseline condition, enhanced target-P3b amplitude and reduced RTV for both ADHD and non-ADHD control. However, the fast-incentive condition also relatively enhanced CNV amplitude but only for the non-ADHD controls and not for the ADHD group. Taken together, such findings on CNV suggest that response preparation is not flexibly adjusted in varying task contexts for ADHD, even in the presence of additional incentives. Similarly, another study using a similar age group reported reduced target-P3b amplitude in the baseline condition and reduced CNV amplitude in the fast-incentive condition in ADHD persisters relative to both ADHD remitters and non-ADHD controls, with these components not distinguishing remitters from non-ADHD controls (James et al., 2017). Thus, such ERP components in the fast task may represent candidate markers of current ADHD status.

##### 3.1.1.2 EEG oscillations

One study assessing EEG power (Table 1B) across the whole duration of the CPT-OX task showed that ADHD persisters, relative to ADHD remitters and non-ADHD controls, had enhanced delta power, both when ADHD diagnosis was determining by parental reports (Cheung et al., 2016) and by self-reports (Du Rietz et al., 2016). Another study reanalysed data generated from a visual oddball paradigm – initially analysed as conventional averaged ERPs (Iacono et al., 2002) – by applying more advanced data reduction techniques to separate underlying components that comprise the ERPs (Gilmore et al., 2011). The technique is known as time-frequency principal component analysis (TF-PCA). It consists of first applying time-frequency analyses (Table 1B) to the same time window as the ERP – in this case the target-P3b ERP – and then applying PCA to isolate overlapping component oscillations based on their distinct frequency features (for more details of this method see Bernat et al., 2008, 2005). These analyses resulted in five principal components (PC) in the delta frequency band (0–2.5 Hz) accounting for 77% of the target-P3b variance. While each of these PCs distinguished controls from those with ADHD at age 17, it also distinguished controls from those with other external psychopathologies, and thus such findings may not be ADHD-specific.

Two studies using oscillatory approaches in tasks relevant to attentional processing focused on very-low frequency (VLF) oscillations (0.02-0.20 Hz) (Table 1B). It has been proposed that VLF oscillations are characteristic of the default mode network (DMN), which is most active during resting conditions and is thought to relate to an interconnected region including the anterior and posterior cingulate cortex, medial prefrontal lobe and precuneus (Broyd et al., 2011). However, VLF EEG power does not appear to distinguish ADHD from non-ADHD controls, at least in young adults. For instance, one study found that VLF EEG power was attenuated from rest to other cognitive states (either a cognitive task or an active waiting period) in a similar fashion for both ADHD and non-ADHD controls at age 22 (Hsu et al., 2013). An additional study also found that VLF EEG power was attenuated from rest to an attentionally-demanding task for both ADHD and non-ADHD controls at age 21 (Broyd et al., 2011). However, the latter study identified that the sources of reduction in VLF EEG power differed between groups, as identified by a proposed source location method for EEG data known as standardized low-resolution brain electromagnetic tomography (s-LORETA) (Pascual-Marqui et al., 1994). More specifically, rest-to-task reduction of VLF EEG power was most prominent in the medial prefrontal cortex for non-ADHD controls but instead in the temporal lobe for ADHD, suggesting that ADHD does not ‘turn off’ typical DMN regions during cognitive tasks. While such findings are preliminary and warrant further replication, they nevertheless suggest that source-based – rather than scalp-based methods in EEG data – can further identify the specific pathophysiology for conditions such as ADHD.

#### 3.1.2 ASD

##### 3.1.2.1 ERP

Selective attention in ASD samples has also employed oddball ERP paradigms. One study using an auditory oddball paradigm suggests that ASD is associated with atypical perceptual processing, particularly increased perceptual capacity (Karhson and Golob, 2016). In non-ASD controls, target-P3b latency increased with high versus low perceptual load, which was manipulated by higher similarity between targets and non-target sounds (thus presumably increasing task difficulty). However, target-P3b latency did not increase with perceptual load in individuals with ASD at age 22, suggesting that they have higher perceptual load capacity, thus remaining ‘unaffected’ by the increasing load.

Another study using the visual oddball paradigm showed that ASD at age 17 was associated with both higher amplitude of frontal P3a and longer latencies of centro-parietal P3b to nontargets, which suggest that ASD is associated with atypical distractor processing (Sokhadze et al., 2009a). Similarly, one study using a feature-based target detection task also found evidence for atypical distractor processing in ASD. Participants had to identify on each trial whether a stimulus was the correct target presenting all the necessary visual features of shape, colour and orientation (Milne et al., 2013). Some trials presented distractor stimuli instead, which could be either relevant or irrelevant: relevant distractors shared both colour and orientation with the target, whereas irrelevant distractors did not. Higher levels of ASD traits at ages 18–27 were associated with a bigger P3b amplitude in response to irrelevant distractors, but not in response to relevant distractors. Thus, such a pattern suggests that ASD is linked to a reduced capacity for filtering out contextually-irrelevant information.

##### 3.1.2.2 EEG oscillations

One study was identified which applied oscillatory approaches to studying attention processing in ASD. Event-related gamma activity (stimulus-locked EEG activity at 30–80 Hz, widely associated with top-down attentional processing and object perception; see Table 1B) was examined using a visual oddball paradigm as described above and initially analysed using ERPs (Sokhadze et al., 2009a). A unique feature of this paradigm is the use of Kanizsa figures as stimuli, which are images depicting a form of optical illusion in which illusory contours, rather than real contours, create the subjective perception of a figure. Higher gamma power was found in ASD in response to nontargets in the left frontal, bilateral parietal and occipital scalp channels. While such scalp-based findings were interpreted as indicating increased competing activity in multiple local networks when processing nontarget stimuli (Sokhadze et al., 2009a), source-based analyses are required to draw firmer conclusions.

### 3.2 Inhibitory control

Seven articles primarily investigated inhibitory control, referring to the selection of the correct response among various competing options. All of these studies focused on individuals with ADHD only, and all considered clinical or research diagnosis. Further, only studies on ERPs were identified. Relevant cognitive paradigms include the stop-signal tasks and go/no-go tasks (see Table 2B). One of these articles (Groom et al., 2008) was also described in the Section 3.1 above on attention processing.

**Table 2B:** Studies on ADHD or ASD using EEG-imaging to investigate of *inhibitory control*.

Study	Clinical group	Diagnosis or trait	Age: Mean (SD)	Age range (years)	Sample size(s)	Comparison group(s) (sample size)	Cognitive paradigm	Analytical method	Features analysed
MacLaren et al., 2007	ADHD	D	20.6 (3.3)	17 to 30	20	Non-ADHD (20)	Stop-signal	ERP	N2, P3b
Woltering et al., 2013	ADHD	D	25.0 (5.8)	–	65	Non-ADHD (32)	Go/no-go	ERP	N2. P3b
Fisher et al., 2011	ADHD	D	24.6 (3.9)	18 to 30	14	Non-ADHD (14)	Go/no-go	ERP	N2, P3b
Shahaf et al., 2012	ADHD	D	24.6 (3.9)	18 to 30	13	Non-ADHD (13)	Go/no-go	ERP	P1, N1, P2, N2
Burden et al., 2010	ADHD	D	19.4 (0.5)	–	15	ALC (16), ADHD + ALC (15), controls (32)	Go/no-go	ERP	P3b
Rodriguez and Baylis, 2007	ADHD	D	19.5 (1.9)	18 to 24	ADHD-C:16; ADHD-IA: 16; ADHD-HI: 16	Non-ADHD (16).	Go/no-go	ERP	P3b
Gorman Bozorgpour et al., 2013	ADHD	D	ADHD-C: 23.2 (1.1); ADHD-IA 24.0 (1.2)	–	ADHD-C: 22; ADHD-IA: 18	Non-ADHD (38)	Go/no-go	ERP	LPR

*Note*. EEG = electroencephalography; ADHD = attention deficit/hyperactivity disorder; ASD = autism spectrum disorder; D = diagnosis; T = trait; ERP = event related potentials; ALC = alcohol-exposure (ALC) only in childhood; ADHD-C = ADHD combined subtype; ADHD-IA = ADHD inattentive subtype; ADHD-HI = ADHD hyperactive/impulsive subtype; LPR = lateralised potential readiness; an additional relevant study by Groom et al., 2008 is presented in Table 1A.

#### 3.2.1 ADHD

##### 3.2.1.1 ERP

Stop-signal tasks refer to cognitive tasks in which a stop signal is embedded into the task, requiring participants to inhibit any response they had previously been instructed to make, for example, to inhibit response to a target. One study using this task found that ADHD at age 21 were associated with reduced amplitude in the N2-P3 complex as well as a delayed P3b latency to the stop-signal, which are known markers of poor response inhibition (MacLaren et al., 2007).

Inhibitory control has also been investigated using variants of the go/no-go task, in which participants have to respond to certain stimuli while inhibiting their response to others. One study found that both ADHD and schizophrenia (but not being sibling of someone with schizophrenia) at age 16 were associated with reduced no-go-N2 amplitude (Groom et al., 2008), suggesting that N2 is a marker of general psychopathology rather than familial risk; however, no-go-P3b effects were not found. Another study found reduced no-go-N2 and reduced no-go-P3b amplitudes in ADHD at age 25 (Woltering et al., 2013). N2 effects were linked to lower accuracy, thus potentially reflecting poor response control (see Table 1A), whereas P3b effects were linked to more inattention symptoms, likely to reflect inefficient attentional allocation.

An auditory go/no-go paradigm also showed that ADHD at 25 was associated with reduced no-go-P3b amplitude and delayed no-go-N2 and no-go-P3b (Fisher et al., 2011). Unexpectedly, ADHD was also associated with more omission errors (not responding to go-trials) and enhanced go-N2. Thus, ADHD-associated deviations in both go ERPs, in addition to no-go ERPs, suggest wider regulation problems beyond inhibitory control. Relevant to diagnosis, a classification approach on these same no-go trials data revealed 92% specificity and 84% sensitivity in discriminating ADHD from non-ADHD controls (Shahaf et al., 2012). Specifically, a network pattern involving a P3b in the delta range at frontal-central-parietal electrodes emerged for non-ADHD controls, whereas a pattern involving early ERP components (P1, N1, P2, N2) in the theta and alpha ranges at central electrodes emerged for ADHD. These patterns suggest that inhibitory deficits typically observed in ADHD are linked to earlier sensory processing (see more on sensory processing later in Section 3.6). Unfortunately, such analyses were not reported for go-trials ERPs, and leaves open whether inclusion of these could improve diagnosis classification further.

One study using a go/no-go paradigm found that young adults at age 19 with a childhood diagnosis of ADHD, and also without a history of prenatal alcohol exposure, showed reduced no-go P3b amplitude, but this was not the case for young adults with a childhood diagnosis of ADHD but with a history of prenatal alcohol exposure (Burden et al., 2010). Another preliminary study investigated subtypes of ADHD with a go/no-go paradigm. It was found that all ADHD subtypes (inattentive, hyperactive and combined) at age 20 showed reduced P3a amplitude (obtained by a difference wave between no-go versus go trials) relative to non-ADHD controls when the instruction was to *inhibit* response to the target. The pattern of findings was slightly different when the instruction was instead to *respond* to the target: all subtypes also showed an attenuated target-P3a amplitude, with the exception of those with the combined subtype (Rodriguez and Baylis, 2007). Such findings suggests that target-P3a in variants of this paradigm can inform research into potential ADHD subtypes.

A separate study however did not find atypical profiles of P3 nor N2 amplitudes in any of the ADHD subtypes (inattentive or combined) at ages 23–24 in a go/no-go task (Gorman Bozorgpour et al., 2013). Instead, those with the inattentive and combined subtypes showed reduced amplitude of the *early* lateralized readiness potential (LRP) for no-go trials, thought to index reduced response preparation (Smulders and Miller, 2012) (see Table 1A). The same groups also showed earlier LRP onsets for no-go trials of increased difficulty (trials demanding discrimination of perceptually similar stimuli), suggesting earlier response preparation in these groups. In addition, the combined subtype showed reduced *late* LRP, suggesting that this individuals in this group were less likely to get ready for the next trial.

#### 3.2.2 ASD

No studies were identified which investigated inhibitory control in ASD in young adults using EEG-imaging, using either ERPs or oscillatory approaches.

### 3.3 Performance monitoring

Nine articles have investigated performance monitoring, defined as a set of cognitive processes implicated in the monitoring of ongoing behaviour to inform response selection and adjustment to achieve optimal goal-directed behaviour (Ullsperger et al., 2014). Such tasks may include aspects of error processing and/or feedback processing. Five of these studies considered clinical or research diagnosis. In total, six studies of performance monitoring focused on ADHD and three on ASD in young adulthood. Cognitive paradigms of performance monitoring in these studies include arrow flanker tasks (McLoughlin et al., 2014b, 2009), go/no-go tasks (Groom et al., 2010; O’Connell et al., 2009), and incentive delay tasks (Boecker et al., 2014; Broyd et al., 2012) (See Table 2C).

**Table 2C:** Studies on ADHD or ASD using EEG-imaging to investigate *performance monitoring*.

Study	Clinical group	Diagnosis or trait	Age: Mean (SD)	Age range (years)	Sample size(s)	Comparison group(s) (sample size)	Cognitive paradigm	Analytical method	Features analysed
Michelini et al., 2016	ADHD	D	ADHD-P: 18.3 (3.0); ADHD-R: 18.9 (3.6)	–	ADHD-P: 87; ADHD-R: 23	Non-ADHD (169)	Flankers task	ERP	N2, ERN, Pe
Chang et al., 2009	ADHD	D	23.7 (3.7)	18 to 30	36	Non-ADHD (32)	Flankers task	ERP	ERN, Pe
Groom et al., 2010	ADHD	D	16.2 (0.3)	14 to 17	23	Non-ADHD (19)	Go/no-go	ERP/oscillations	ERN, Pe, theta, ITC
O’Connell et al., 2009	ADHD	D	23.7 (5.1)	–	18	Non-ADHD (21)	Go/no-go	ERP	ERN, early positivity, Pe
Broyd et al., 2012	ADHD	T	21.8 (4.5)	–	18	–	Incentive delay task	ERP	CNV, LPP
Boecker et al., 2014	ADHD	T	24.4 (-)	–	162	–	Incentive delay task	ERP	CNV
Carter Leno et al., 2016	ASD	T	22.0 (4.4)	18 to 52	16	Low traits (15); Psychopathic traits (23)	Feedback paradigm	ERP	FRN
Cox et al., 2015	ASD	T	23.9 (3.4)	18 to 35	17	Low traits (18)	Incentive delay task	ERP	P3b
Thillay et al., 2016	ASD	D	21.4 (-)	18 to 27	12	Non-ASD (12)	Target detection task	oscillations	N1, P2, N2, mu

*Note*. EEG = electroencephalography; ADHD = attention deficit/hyperactivity disorder; ASD = autism spectrum disorder; D = diagnosis; T = trait; ERP = event related potentials; ADHD-P = ADHD persisters; ADHD-R = ADHD remitters; ERN = error-related negativity; Pe = error positivity; ITC = inter-trial coherence; CNV = contingent negative variation; LPP = late positive potential; FRN = feedback-related negativity.

#### 3.3.1 ADHD

##### 3.3.1.1 ERP

Flankers tasks (using stimuli such as arrows or letters) typically consist of congruent trials, where flankers and targets are compatible, and incongruent trials, where flankers and targets are incompatible. ERPs associated with this task include the N2, which is increased in incongruent trials and is thus thought to index ‘conflict monitoring’, or processing of high conflict stimuli that induce potential competing responses (Table 1A). Two additional error-related ERPs are relevant: the error-related negativity (ERN) which is thought to index general ‘unconscious’ error processing, and error-related positivity or Pe, which is believed to reflect conscious error processing informing adjustment of response strategy (Nieuwenhuis et al., 2001; Wessel, 2012) (Table 1A).

Two studies employed flanker tasks in young adult samples with ADHD. Using an arrow flanker task (McLoughlin et al., 2014b), a study that followed up individuals with childhood ADHD found that ADHD persisters at age 18–19 (i.e., those who persisted with the disorder into adulthood) showed reduced ERN and Pe, in comparison to ADHD remitters (i.e., those who remitted the diagnosis). However, both ADHD persisters and remitters showed reduced N2 amplitude (Michelini et al., 2016). Such findings suggest that ADHD remission is linked to a typical pattern of sensitivity to errors, but with atypical processing in conflict monitoring despite the improvement in behavioural symptoms. However, when IQ was accounted for, not only ADHD persisters but also remitters showed reduced ERN and Pe. Such findings suggest that higher IQ in ADHD remitters allows for compensation in adulthood, in that IQ supports an ability in ADHD remitters to process errors to adjust performance. Another cross-sectional study, using the Eriksen flanker task (Eriksen and Eriksen, 1974), also showed that ADHD at age 23 was associated with reduced ERN amplitude and also shorter ERN latency, but with typical profiles of Pe amplitude and latency (Chang et al., 2009). It is possible that findings with error-related ERPs in ADHD depends on the specific time point within young adulthood (e.g., ages 18 vs. 23).

Error processing has also been investigated using a go/no-go paradigm, in which participants are typically required to respond to one type of stimuli (e.g., ‘X’) but withhold response to another type (e.g., ‘K’). For instance, ADHD at age 16 (Groom et al., 2010) was associated with a typical profile of ERN amplitude – in stark contrast to findings from studies using variants of flanker tasks as described above (Chang et al., 2009; Michelini et al., 2016) – and only a statistical trend of a reduced Pe amplitude. A similar pattern was shown by another study using a go/no-go task, which consisted of serially presented word stimuli of different colours (O’Connell et al., 2009). Participants were instructed to respond to each stimulus unless the same stimulus was immediately repeated (repeat no-go) or it contained a word that did not match its colour (incongruent no-go). A critical difference to other paradigms is that participants were also told to press an ‘awareness’ button whenever they believed they had made an error, allowing for classification of aware versus unaware errors. ADHD at age 24 in this study was also associated with a typical profile of ERN amplitude, but alongside a pattern of reduced Pe amplitude, more errors committed and less awareness of them. Thus, ERP findings on error-related processing in ADHD may depend on the specific paradigm employed.

Additional studies based on ADHD traits investigated performance monitoring in the context of feedback and reward. In one study, participants were asked to respond to a target stimulus under two conditions: either a fast response would lead to both positive feedback and immediate start of the next trial avoiding a delay (escape condition), or instead a fast response would not influence the delay of the next trial at all (no-escape condition). Higher number of ADHD symptoms at age 21 was associated with larger differences in CNV amplitude between the escape versus no-escape conditions, which could be interpreted as reflecting greater response preparation to conditions in which delayed rewards could be avoided (Broyd et al., 2012).

Reward processing was also investigated in a large sample from an epidemiological longitudinal cohort at age 24 (Boecker et al., 2014). In a monetary-incentive delay task, participants were instructed to respond quickly to potentially rewarding signals: a happy smiley face signalling monetary reward or a scrambled smiley face signally verbal reward (‘fast reaction!’) only. While enhanced CNV (indexing response preparation) was found for anticipation of monetary reward compared to verbal reward, such enhancement was not associated with lifetime ADHD symptoms based on a composite score across five longitudinal assessments (Boecker et al., 2014). While such trait-based studies require replication in clinical samples, it is possible that greater response preparation in ADHD is linked to the specific form of reward in a given paradigm.

##### 3.3.1.2 EEG oscillations

A study described previously using a go/no-go task (Groom et al., 2010) also examined oscillatory activity in relation to errors, using the same time windows post-error as employed for the analysis of ERN (-50 to 100 ms; early window) and Pe (100 to 350; late window). Evoked power and inter-trial coherence (ITC) in the theta range (4–7 Hz) were considered (see Table 1B) based on previous findings that they may largely account for the ERN (e.g., Luu et al., 2004). ADHD at age 16 was associated with reduction in late evoked theta power and also with reduction in both early and late theta ITC. Such findings suggest that ADHD is linked to impaired error processing potentially arising from reduced efficiency of coordination of brain activity. Of note, such oscillatory measures were able to reveal more reliable differences between ADHD and controls, compared to ERP measures of ERN or Pe amplitudes within the same study (Section 3.3.1.1).

#### 3.3.2 ASD

##### 3.3.2.1 ERP

Performance monitoring in ASD has been examined using different tasks from those employed in the ADHD literature. For instance, one study examined neural response to different types of feedback: social (verbal), non-social (candy) and neutral (shape), using a paradigm in which participants were asked to seek positive feedback or avoid negative feedback (Carter Leno et al., 2016). Individuals with high ASD traits at age 22 showed reduced FRN amplitude in social contexts only, suggesting reduced saliency of social feedback. Using a similar paradigm, another study found that individuals with high versus low ASD traits at age 23 did not show differences in neural processing of non-social and non-reward feedback (Cox et al., 2015). However, those with high ASD traits showed reduced P3b amplitude specifically to social reward feedback. Such findings suggest that ASD traits modulate neural reward sensitivity but mainly within social contexts.

A key aspect of monitoring performance is the ability to make predictions in uncertain environments. One preliminary study examined predictive processing using a visual target detection task in ASD at age 21 (Thillay et al., 2016). Both ASD and non-ASD groups showed faster RT for predictable compared to random sequence targets, as well as enhanced P3b amplitude and enhanced CNV amplitude for the predictable targets. However, only ASD was associated with faster absolute RT, increased CNV amplitude and shorter N2 latency for the random targets, suggesting that ASD is associated with increased response preparation to stimuli even in a random context. It is also possible that such atypical processing of random stimuli relates to the preference for ‘sameness’ in ASD.

##### 3.3.2.2 EEG oscillations

The study above also examined the relevance of mu rhythms in prediction processing (Thillay et al., 2016). Mu rhythms refer to oscillations in the alpha frequency range (8–14 Hz), typically recorded over the sensorimotor cortex (from left central electrodes) (Table 1B). Reduction in the power of mu rhythms is thought to index motor anticipation (mu rhythms are also relevant for the domain of imitation and empathy, which we elaborate in a later section of the review in Section 3.5). Non-ASD controls showed an expected decrease in mu power *before* predictive targets compared to random targets; however, this decrease was not found for ASD at age 21, suggesting a motor anticipation failure. This deficit was accompanied by a reduced benefit in RTs to predictable targets in individuals with ASD. In contrast, ASD was associated with increased alpha activity over frontocentral regions *before* the onset of predictable targets compared to random targets. Taken such findings together, ASD appears to be associated with a profile of reduced motor preparation to predictable targets, as reflected by findings on mu oscillations, alongside compensatory strategies to counteract such lack of motor preparation, reflected by findings on frontocentral alpha oscillations.

### 3.4 Face processing

Thirteen studies investigated face processing using EEG-imaging. Only two studies did not consider clinical or research diagnosis. Whereas two studies focused on ADHD, 11 focused on ASD. The cognitive paradigms employed examined various aspects of face processing, including face orientation, gaze direction, mouth shape, and comparisons to non-face stimuli and verbal stimuli of names. One of the studies in ASD focused on name processing only, but is included in this section due to its relevance to potential shared mechanisms with face processing (see Table 2D).

**Table 2D:** Studies on ADHD or ASD using EEG-imaging to investigate *face processing*.

Study	Clinical group	Diagnosis or trait	Age: Mean (SD)	Age range (years)	Sample size(s)	Comparison group(s) (sample size)	Cognitive paradigm	Analytical method	Features analysed
Raz and Dan, 2015a	ADHD	D	24.1 (1.7)	–	17	Non-ADHD (20)	Visual oddball with faces	ERP	P1, N170, P3b
Raz and Dan, 2015b	ADHD	D	25.4 (2.1)	–	21	Non-ADHD (19)	Visual oddball with faces	ERP	P3b, N170, N3
O’Connor et al., 2005	ASD	D	24.6 (8.8)	18 to 45	15	Non-ASD (15)	Face/emotion processing	ERP	P1, N170
O’Connor et al., 2007	ASD	D	23.5 (5.2)	18 to 41	15	Non-ASD (15)	Face/object processing	ERP	P1, N170
Stavropoulos et al., 2016	ASD	T	22.7 (1.7)	–	20	–	Face/emotion processing	ERP	P1, N170, P3b
Magnée et al., 2011	ASD	D	22.7 (3.8)	–	23	Non-ASD (24)	Multisensory integration	ERP	P2, N170
Webb et al., 2010	ASD	D	22.4 (6.1)	18 to 44	29	Non-ASD (28)	Face/object processing	ERP	P1, N170, P2, N250, face-N400
Webb et al., 2012	ASD	D	23.1 (6.9)	18 to 44	32	Non-ASD (32)	Face/object processing	ERP	P1, N170
Faja et al., 2016	ASD	D	23.3 (7.7)	18 to 45	27	Non-ASD (25)	Face/emotion processing	ERP	P1, VPP, N170, EPN
Cygan et al., 2014	ASD	D	–	17 to 27	23	Non-ASD (23)	Face/name processing	ERP	P1, N170, P3b
Nowicka et al., 2016	ASD	D	19.3 (2.4)	17 to 23	15	Non-ASD (15)	Name processing	ERP/oscillations	P3b, alpha, beta
Lassalle and Itier, 2015	ASD	T	20.4 (2.3)	18 to 29	22	Low traits (46)	Face/gaze processing	ERP	P1, EDAN, ADAN
Jaime et al., 2016	ASD	D	16.2 (2.3)	–	16	Non-ASD (17)	Joint attention task	oscillations	alpha, beta

*Note*. EEG = electroencephalography; ADHD = attention deficit/hyperactivity disorder; ASD = autism spectrum disorder; D = diagnosis; T = trait; ERP = event related potentials; VPP = vertex positive potential; EPN = early posterior negativity; EDAN = early directing attention negativity; ADAN = anterior directing attention negativity.

#### 3.4.1 ADHD

##### 3.4.1.1 ERP

Those with ADHD appear to demonstrate atypical neural processing to facial stimuli. Two studies embedded face stimuli within a visual oddball paradigm. One study found that ADHD at age 24 was associated with enhanced target-P1 amplitude (Table 1A) to emotional faces (angry and happy), suggesting enhanced initial perceptual encoding for emotional stimuli (Raz and Dan, 2015a). However, the amplitude of the N170 (Table 1A), specific to face processing, was enhanced for angry faces only. Non-ADHD controls had the reversed pattern of enhanced N170 for happy faces only. Thus, ADHD appeared to be associated with enhanced processing of angry faces despite the indication of enhanced attentional orienting to emotional stimuli in general. Another study using *only* angry faces did not find the same N170 effects, but instead showed that ADHD at age 24 was associated with enhanced target-P3b amplitude and reduced target-N3 amplitude (also known as LPN or late posterior negative slow wave) in response to angry faces, which was interpreted as an index of more effortful processing (Raz and Dan, 2015b) (Table 1A).

#### 3.4.2 ASD

##### 3.4.2.1 ERP

Given that impairment in social cognition is thought to be a core feature of ASD, it is not surprising that a large number of ERP studies in this disorder have focused on face processing given that facial information is used for everyday communication. Various studies have linked ASD with atypical facial processing. For instance, ASD at 24 was associated with relatively longer P1 and N170 latencies as well as reduced N170 amplitude in response to faces depicting either happy, sad, angry, scared or neutral expressions (O’Connor et al., 2005). ASD at age 23 was also associated with shorter N170 latencies to whole faces as well as to face parts (eyes or mouths), but not to objects (O’Connor et al., 2007). Another study found that higher ASD traits at age 23 were correlated with longer P1 latency and longer N170 latency for both conscious face processing (stimulus presented for 200 ms, i.e., long enough for awareness of perception) and nonconscious face processing (stimulus presented for 16 ms, i.e., too briefly for awareness of perception) (Stavropoulos et al., 2016). Taken together, such studies suggest that ASD is associated with reduced ability to process information holistically relevant to facial stimuli, as indexed by findings on N170 (Table 1A).

The pattern in relation to N170 may also extend to other modalities. For example, non-ASD controls exhibited a larger N170 amplitude to emotionally congruent face-voice pairings (e.g., happy face with happy laugh) compared to incongruent face-voice pairings (e.g., happy face with gasping sound), whilst such a congruency effect was not observed in ASD at age 22 (Magnée et al., 2011).

Atypical patterns of ERP findings in relation to face processing in ASD, however, are not always found in young adult samples. One study failed to find group differences between ASD at age 23 and non-ASD controls in ERPs locked to face stimuli, such as P2 and N250 in response to familiar versus unfamiliar faces, and N400 in response to repeated versus novel faces (Webb et al., 2010). It is possible that such ERPs may index other processing aspects in relation to face stimuli not captured by the N170 ERP.

ASD-linked atypical profiles of N170, however, are not always consistent. For example, a study using house and face stimuli failed to replicate group differences in N170 between ASD and non-ASD controls at age 22 (Webb et al., 2012). Another study using open and closed mouth emotional faces also failed to find ASD-linked atypical profiles in N170 at age 23 (Faja et al., 2016). Instead, this latter study found ASD atypical profiles in the early posterior negativity (EPN), an enhanced negative-tending amplitude measured at 200–350 ms, thought to reflect perceptual attention underlying coding and recognition of facial expressions in the occipital and temporal cortex (Citron, 2012) (Table 1A). First, EPN ERP in the ASD group showed a more right lateralised EPN scalp distribution relative to the non-ASD group. Second, while there were differences in EPN amplitude between faces with open mouths and faces with closed mouths in the non-ASD group, such differences were absent in ASD. Taken together, these findings suggest that individuals with ASD also rely on different cues for processing emotional faces, not captured by the N170 ERP.

Two studies investigated the neural correlates of both name and face processing in ASD. One study compared attention to self-related stimuli (own + close other’s names and faces) to control stimuli (famous + unknown names and faces) (Cygan et al., 2014). Individuals with non-ASD showed higher P3b amplitude to self-related stimuli relative to the control stimuli, thus showing a self-preference effect across names and faces. In contrast, ASD at age 17–27 showed enhanced P3b amplitude to faces across all face types relative to all names, thus without such a self-preference effect. A subsequent study also found a self-preference effect in non-ASD controls at age 19 showing enhanced P3b to own name versus close-other’s name (Nowicka et al., 2016); however, this self-preference P3b effect was absent in ASD.

One study investigated the effect of gaze orienting on emotional facial expressions. The paradigm involved initial presentation of an individual’s face with neutral expression and with straight gaze (i.e., direct gaze-at); the gaze then changes to either a right- or leftward gaze (i.e., non-gazed-at or averted), followed by the same individual with an either happy or fearful expression (Lassalle and Itier, 2015). Unlike for individuals with low ASD traits, an enhanced target-P1 amplitude for direct gaze-at faces (compared to averted gaze) was not observed for those with high ASD traits at age 20, suggesting a reduction in attention to direct-gaze faces in ASD. Two additional ERP components were investigated in this study: the early directing attention negativity (EDAN), occurring at posterior electrodes between 200–300 ms after cue presentation and which is associated with attention-orienting, and the anterior directing attention negativity (ADAN), occurring at anterior electrodes between 300–500 ms after cue presentation and which is associated with attention-holding (see Table 1A). High ASD traits at age 20 were associated with reduced amplitude in both the EDAN and ADAN to happy faces but not fearful faces. This has been interpreted to indicate that ASD traits influence the reward value of happy faces or the degree of avoidance of stimuli signaling invitation to social interactions.

##### 3.4.2.2 EEG oscillations

Name processing has also been examined in relation to brain connectivity (Nowicka et al., 2016), which was investigated both in terms of a measure of coherence, indexing synchronization between two signals (with high coherence proposed to indicate greater functional integration across the cortex) (Table 1B), and a measure of directed transfer function (DTF), thought to index the strength and direction of the activity flow between the locations (with high DTF indicating greater connectivity). ASD at age 19 was associated with a pattern of atypical functional connectivity during a name recognition task where participants were presented with self-, close-other’s, famous, and unknown names. This pattern involved decreased coherence within the beta band: lower DTF values (under-connectivity) for the long-range connections, alongside higher DTF values (over-connectivity) for the local connections. Moreover, another study found an overall decrease in temporal-central alpha coherence in the right hemisphere in ASD at age 16 during a joint attention task, in which a human model either gazed or did not gaze in the direction of a dot (Jaime et al., 2016). A positive correlation was also found between degree of coherence and social cognitive performance in non-ASD controls but not for ASD. Such a pattern of results suggests that altered connectivity in the alpha band gives rise to joint attention impairments.

### 3.5 Imitation and empathy

Six articles investigated processing relevant to behavioural imitation and empathy. All of these studies focused on ASD only, and considered clinical or research diagnosis. The focus on ASD may be driven by theoretical perspectives linking deficits in imitation with aspects of social cognition that are thought to be ‘disrupted’ in ASD, such as difficulties with empathy (e.g., Iacoboni, 2009). Cognitive paradigms employed typically aimed to measure processing of non-emotional and emotional actions (e.g., pain being inflicted) (see Table 2E).

**Table 2E:** Studies on ADHD or ASD using EEG-imaging to investigate *imitation and empathy*.

Study	Clinical group	Diagnosis or trait	Age: Mean (SD)	Age range (years)	Sample size(s)	Comparison group(s) (sample size)	Cognitive paradigm	Analytical method	Features analysed
Fan et al., 2014	ASD	D	20.4 (4.3)	16 to 29	20	Non-ASD (20)	Visual stimuli task: pain	ERP/oscillations	N2, LPP, mu
Chien et al., 2017	ASD	D	20.5 (5.2)	–	31	Non-ASD (22)	CHEP paradigm	ERP	P2, N2
Althaus et al., 2015	ASD	D	22.7 (4.8)	18 to 31	31	Non-ASD (30)	Emotional picture task	ERP	LPP
Bernier et al., 2007	ASD	D	23.6 (4.9)	–	14	Non-ASD (15)	Mature imitation task	oscillations	mu
Oberman et al., 2005	ASD	D	16.6 (13.0)	6 to 47	10	Non-ASD (10)	Hand movement task	oscillations	mu
Fan et al., 2010	ASD	D	17.7 (4.5)	11 to 26	20	Non-ASD (20)	Hand movement task	oscillations	mu

*Note.* EEG = electroencephalography; ADHD = attention deficit/hyperactivity disorder; ASD = autism spectrum disorder; D = diagnosis; T = trait; ERP = event related potentials; LPP = late positive potential; CHEP = contact heat-evoked potentials.

#### 3.5.1 ADHD

No studies were identified which investigated imitation and empathy in ADHD in young adults using EEG-imaging, using either ERPs or oscillatory approaches.

#### 3.5.2 ASD

##### 3.5.2.1 ERP

One study used ERPs to investigate the perception of pain experienced by others (Fan et al., 2014), and found distinct ERP profiles between ASD at age 20 and non-ASD controls. Only ASD was associated with increased N2 amplitude in response to stimuli depicting solo pain (i.e., single person experiencing accidental pain, such as someone slamming a car door onto their hand) compared to solo no-pain (i.e., single person in a non-painful situation) as well as increased N2 amplitude in response to stimuli depicting dyad pain (i.e., a person in pain caused by another person) compared to those depicting dyad non-pain (i.e., two people in a non-painful scenario). In contrast, only non-ASD controls showed a more positive deflection in late positive potential (LPP) (Table 1A) in response to stimuli depicting dyad pain versus those depicting dyad non-pain. Furthermore, reduced LPP amplitude was associated with more autistic traits but a lower pain threshold. Taken together, these findings suggest that atypical early sensory processing in ASD, as reflected by the N2, can impact on later cognitive processing relevant to empathy, as reflected by the LPP. The authors argued that the stronger N2 in response to all pain-related stimuli, along with the attenuated LPP in response to stimuli depicting dyad-pain specifically, reflects a potential dissociation in ASD between affective arousal (feeling distress) and social understanding (understanding potential sources of the distress).

Further support for atypical early pain processing in ASD at age 20 was demonstrated by a reduced P2 amplitude induced by thermal pain, administered with a contact heat-evoked potential stimulator on the right lateral leg, despite equivalent self-reported pain levels to non-ASD controls (Chien et al., 2017). Administration of oxytocin appeared to enhance the LPP amplitude in individuals with ASD at age 22, but only if they were also easily distressed when seeing others in stressful situations (Althaus et al., 2015), suggesting that importance of considering individual differences within ASD.

##### 3.5.2.2 EEG oscillations

A series of studies has examined the mu rhythm in ASD. Mu suppression has been proposed to reflect activity in the human mirror neuron system (Fox et al., 2016) (Table 1B), although this functional interpretation remains highly controversial and there is disagreement in the literature (see a review see Hobson and Bishop, 2017).

There is some evidence of an atypical profile of mu suppression within ASD in paradigms investigating action execution and action observation. For example, similar to non-ASD controls, ASD was associated with mu suppression during hand movement *execution*; however, contrary to non-ASD controls, reduced mu suppression during hand movement *observation* was found for ASD at age 23 (Bernier et al., 2007). Similarly, a lack of mu suppression during action *observation* was found for ASD in a sample with mean age of 16, (Oberman et al., 2005). While such preliminary findings have been interpreted as a deficit in the mirror neuron system in ASD, a more recent study did not observe such mu suppression differences for *execute* nor *observe* conditions between ASD and non-ASD controls at age 17 (Fan et al., 2010).

As some studies have linked mu suppression with ‘mirroring’ others motor actions, another study examined whether it extends to ‘mirroring’ of others’ emotions, such as perceiving others’ pain. The study already described above using ERPs (Fan et al., 2014) also investigated mu suppression while processing stimuli depicting pain. It was found that both ASD at age 20 and non-ASD controls exhibited stronger mu suppression to stimuli depicting solo pain compared to those depicting solo no-pain. However, both groups differed in relation to processing of stimuli depicting a pair of individuals, which putatively introduced a social context. More specifically, mu suppression was greater for stimuli depicting dyad pain compared to stimuli depicting dyad no-pain for the ASD group, whilst mu suppression for both stimuli type were comparable for the non-ASD group. Such ‘excessive mirroring’ in ASD to pain-relevant stimuli is consistent with the authors’ interpretation of heightened empathic arousal in ASD in the absence of the relevant social understanding. However, such findings may appear contradictory to the pattern, albeit an inconsistent one, of ‘reduced mirroring’ to action observation in ASD in some studies described before (e.g., Bernier et al., 2007). While further replications are required to determine the reliability of these findings, it is also important to bear in mind that the interpretation of findings on mu suppression remains highly controversial, particularly in relation to mirror neurons and empathy processing (cf. Hobson and Bishop, 2017).

### 3.6 Sensory processing

Seventeen articles have investigated sensory processing in ADHD and ASD in young adults. Only two studies did not consider clinical or research diagnosis. Four studies focused on ADHD and thirteen focused on ASD (only two of which examined dimensional traits). The majority of studies on ASD reflects the widely reported hyper- and hypo-sensitivities in sensory processing in ASD which has been recognised as an important feature of ASD in the latest DSM (APA, 2013). Paradigms include those that examine low-level stimulus characteristics such as orientation and brightness, as well as those using more socially-relevant stimuli such as vocal sounds and faces (see Table 2F).

**Table 2F:** Studies on ADHD or ASD using EEG-imaging to investigate *sensory processing*.

Study	Clinical group	Diagnosis or trait	Age: Mean (SD)	Age range (years)	Sample size(s)	Comparison group(s) (sample size)	Cognitive paradigm	Analytical method	Features analysed
Kim et al., 2015	ADHD	D	16.0 (-)	13 to 18	16	Non-ADHD (15)	Colour VEP	ERP	P1
Nazhvani et al., 2013	ADHD	D	16.9 (6.3)	10 to 22	12	BMD (12), controls (12)	Light flash paradigm	ERP	P1
Sable et al., 2013	ADHD	D	–	18 to 23	21	Non-ADHD (18)	Tone processing during film	ERP	P1, N1, P2, N2
Gonen-Yaacovi et al., 2016	ADHD	D	25.0 (-)	21 to 27	17	Non-ADHD (17)	Brightness detection task	ERP/oscillations	P1, N1
Fan and Cheng, 2014	ASD	D	21.5 (3.8)	–	20	Non-ASD (20)	Passive oddball paradigm	ERP	MMN, P3a
Lodhia et al., 2014	ASD	D	22.2 (6.0)	16 to 34	16	Non-ASD (16)	Dichotic pitch paradigm	ERP	P400, ORN
Magnée et al., 2009	ASD	D	22.9 (2.0)	–	13	Non-ASD/SCZ (16), SCZ(13)	Suppression paradigm	ERP	P50, N1, P2
Vandenbroucke et al., 2008	ASD	D	20.8 (4.1)	16 to 28	13	Non-ASD (31)	Discrimination task	ERP	ERP wave 700 ms post-stimuli
Kovarski et al., 2016	ASD	D	21.5 (3.0)	18 to 27	20	Non-ASD (22)	Passive visual task	ERP	N75, P1, N135
Jemel et al., 2010	ASD	D	25.5 (4.6)	18 to 31	16	Non-ASD (14)	Contrast sensitivity task	ERP	P1, N80
Yamasaki et al., 2011	ASD	D	–	20 to 39	12	Non-ASD (12)	Motion detection task	ERP	N170, P2
Magnee et al., 2008	ASD	D	21.1 (4.0)	–	12	Non-ASD (13)	Audio-visual paradigm	ERP	N1, P2
Megnin et al., 2012	ASD	D	16.9 (0.3)	–	14	Non-ASD (14)	Semantic integration task	ERP	N1, P2, N4
Perry et al., 2015	ASD	D	25.0 (1.2)	–	13	Non-ASD (13)	Interpersonal distance task	ERP	P1, N1
Peled-Avron and Shamay-Tsoory, 2017	ASD	T	23.0 (4.1)	18 to 39	29	–	Human/object touch	ERP	P1, LPP
Snijders et al., 2013	ASD	D	22.0 (4.0)	–	12	Non-ASD (12)	Visual stimulation task	oscillations	gamma
Dickinson et al., 2015	ASD	T	25.0 (-)	18 to 45	33	–	Orientation discrimination task	oscillations	gamma

*Note.* EEG = electroencephalography; ADHD = attention deficit/hyperactivity disorder; ASD = autism spectrum disorder; D = diagnosis; T = trait; ERP = event related potentials; VEP = visual evoked potentials; BMD = bipolar mood disorder; MMN = mismatch negativity; ORN = object-oriented negativity; SCZ = schizophrenia; LPP = late positive potential.

#### 3.6.1 ADHD

##### 3.6.1.1 ERP

ADHD has been associated with atypical profiles of sensory processing across various modalities. In a study examining colour perception, ADHD at age 16 was associated with an enhanced P1 amplitude to blue-yellow chromatic stimuli, but typical P1 amplitude for red-green stimuli and typical performance on an ophthalmological exam (Kim et al., 2015). P1-related findings may also have implications for diagnosis, as a statistical technique was able to classify individuals at age 17 with ADHD, bipolar mood disorders and nonclinical controls at 92.9% accuracy, based on combined information of the amplitudes and latencies of P1 elicited by a flash light stimuli (Nazhvani et al., 2013).

Atypical auditory processing has also been identified in ADHD, using a sound perception task. For this task, while watching a silent video, trains of consecutive 5-tones were presented (with onset-to-onset time of 400 ms), separated by either 1 s or 5 s between each train (Sable et al., 2013). ADHD individuals at ages 18–23 did not show the typically large N1 amplitude that is seen in response to salient sounds (thought to be elicited when there is a 5 s delay between trains of sounds), which suggests reduced saliency of these stimuli in ADHD. In addition, ADHD was associated with enhanced N2 amplitude to the sounds, with the authors interpreting such findings as a sign of compensatory increased top-down attention suppression in order to watch the video.

In addition to traditional scalp-based data, one study also used data derived from independent component analysis (ICA), a statistical technique that can separate neural and non-neural sources in the EEG signal thus leading to improved signal-to-noise ratio and source estimation (Jung et al., 2001). ICA was used to perform single-trial analyses of early sensory components (P1 amplitude for visual and N1 amplitude for the auditory stimuli during an incidental bright detection task) (Gonen-Yaacovi et al., 2016). ADHD at age 25 was associated with increased trial-by-trial variability in P1/N1 amplitudes in the time window pre, post and without stimulus, suggesting an ongoing issue with increased neural variability, which can reflect perception of the environment in a less reliable manner.

##### 3.6.1.2 EEG oscillations

The same study focusing on trial-to-trial variability of early-sensory components (Gonen-Yaacovi et al., 2016) also examined ITC of EEG activity to examine the similarity in phase across trials (Table 1B), focusing on both theta (4–8 Hz) and alpha bands (8–12 Hz). ADHD was associated with lower ITC values (i.e., larger variability) for poststimulus-locked EEG, which may indicate that increased neural variability is a potential driver of the observed behavioural variability in ADHD as suggested previously (cf. McLoughlin et al., 2014b). However, this pattern was found across all portions of the EEG, including before and after the stimulus, as well as in trials where the stimulus was omitted. Thus, it appears that ongoing and not just stimulus-locked EEG exhibits variability in ADHD.

#### 3.6.2 ASD

##### 3.6.2.1 ERP

In the auditory domain, discrimination of spoken syllables of varying emotional prosodies were investigated using a paradigm that elicited mismatch negativity (MMN), an ERP component thought to index detection of unexpected stimuli (Garrido et al., 2009) (Table 1A). ASD at age 22 was linked to reduced MMN to non-vocal sounds, as well as a lack of differentiation between MMN responses to vocal happy versus angry syllables (Fan and Cheng, 2014), suggesting atypical processing of emotional voices. In a dichotic pitch paradigm requiring the discrimination of two pitches (Lodhia et al., 2014), ASD at age 22 was linked to poorer discrimination performance and also to reduced amplitude of the object-related negativity, a negative ERP component peaking at 150–250 ms post-stimulus, which has previously thought to index auditory integration (Alain et al., 2001) (Table 1A). These findings suggest a reduced ability to separate dichotic stimuli into spatially separate sound qualities (pitch and noise) in ASD, potentially explaining ASD-linked sensitivity to sensory stimuli. Nevertheless, one study found that repeated presentation of both auditory-then-auditory stimuli pairs (i.e., two consecutive identical sounds) and visual-then-auditory stimuli pairs produced the equivalent reduction in P50 (Magnée et al., 2009), for both ASD at age 22 and non-ASD controls, suggesting a similar degree of sensory gating between both groups (i.e., the ability to filter out redundant sensory information) (Potter et al., 2006) (Table 1A).

Some studies investigated visual processing exclusively. Using a discrimination task (of stimuli made of black line segments), non-ASD controls showed a negative ERP deflection around 120 ms post-stimulus, believed to signal boundary detection (i.e., detecting the boundary between visual stimuli of two different orientation in a given trial). This effect was diminished in ASD at age 20 (Vandenbroucke et al., 2008). Another study showed that ASD was associated with a typical later ERP (215–320 ms) believed to signal surface segregation (i.e., separating a scene into figure and ground), suggesting a preserved and/or compensatory mechanism in ASD in sensory processing.

Additional experiments used visual evoked potentials (VEP), such as P1 and N80, to investigate integrity of the visual pathway: ASD was associated with reduced P1 amplitude in a pattern-reversal paradigm at age 21 (Kovarski et al., 2016); and also with a lack of differentiation of an early ERP (N80 amplitude) between black-and-white gratings stimuli of mid versus high spatial frequency at age 25 (Jemel et al., 2010). Together, these findings link ASD with atypical early sensory processing of low-level perceptual features. A further study suggests that ASD is also associated with atypical processing of visual motion stimuli, particularly those linked to the ventro-dorsal visual stream: ASD at ages 20–39 was associated with longer N170 and P2 latencies to stimuli depicting radial optic flow (dots consistently moving inward or outward from a central point) but not to stimuli depicting horizontal movements (dots consistently moving leftward or rightward) (Yamasaki et al., 2011).

Other studies investigated the integration of different modalities. One study found that both ASD at age 21 and non-ASD controls showed equivalent reductions in amplitude of N1 and reductions in latencies of N1 and P2 in response to audio-visual stimuli relative to unimodal stimuli (Magnee et al., 2008). In contrast to such findings on earlier ERPs, ASD was associated with an absence of a later ERP starting at 500 ms post-stimuli in the frontal electrodes, which typically differentiates congruent versus incongruent audio-visual stimuli (e.g., a face with lips making the movement of ‘ada’ while the auditory stream presents ‘aba’) in non-ASD controls. These findings suggest atypical high-level integration of auditory with visual information in ASD, as reflected by later ERPs, despite typical low-level processing of multimodal features, as reflected by earlier ERPs. Nevertheless, atypical low-level sensory processing was identified in another study investigating processing of spoken words. This study showed that ASD at age 16 was associated with a lack of P2 amplitude enhancement in both visual-only (i.e., face) and audio-visual (i.e., face and voice) presentations (Megnin et al., 2012).

Sensory processing in ASD has also been examined in interpersonal contexts. A study examined neural responses to stimuli representing various levels of interpersonal distance preferences to strangers or friends (Perry et al., 2015). For ASD at age 25, N1 amplitude was correlated with a behavioural measure of interpersonal distance, suggesting that atypical levels of sensory sensitivity can explain some of the ASD-linked difficulties in social domains. Another study found that pictures of interpersonal touch in the high ASD trait group at age 23, relative to the low trait group, elicited equivalent P1 amplitude but larger LPP amplitude, which may index hypervigilance to social touch in ASD particularly at later stages of processing (Peled-Avron and Shamay-Tsoory, 2017) (Table 1A).

##### 3.6.2.2 EEG oscillations

Sensory processing in ASD has been studied in non-social domains using oscillatory measures. Two studies showed patterns of atypical context modulation during early visual perception in ASD. In one of these, participants were presented with Gabor patches with different degrees of orientation (Snijders et al., 2013). It was found that, unlike for non-ASD controls, event-related gamma-power (Table 1B) did not increase with increasing contextual modulation (by increasing the amount of homogeneity in visual orientation of the stimuli) in ASD at age 22. In another study using a similar paradigm, participants were instructed to assess the orientation of the target grating (Dickinson et al., 2015). ASD trait scores at age 25 were associated with two main findings: lower orientation discrimination thresholds (i.e., enhanced perceptual discrimination) and higher peak induced gamma frequency (Table 1B). Both of these findings have been previously associated with increased neural inhibition (Edden et al., 2009), and thus point towards a potential role of neural inhibition in ASD.

### 3.7 Memory and language

Seven articles investigated various processes related to memory, learning and language. Five of the studies considered clinical or research diagnosis. Three of these focused on ADHD and four on ASD. A variety of cognitive paradigms were investigated, which examined short-term or working memory (WM), long-term episodic memory and semantic memory/language processing (see Table 2G).

**Table 2G:** Studies on ADHD or ASD using EEG-imaging to investigate *memory and language*.

Study	Clinical group	Diagnosis or trait	Age: Mean (SD)	Age range (years)	Sample size(s)	Comparison group(s) (sample size)	Cognitive paradigm	Analytical method	Features analysed
Kim et al., 2014	ADHD	D	–	19 to 35	37	Non-ADHD (25)	Delayed match-to-sample task	ERP	P3b
Biehl et al., 2013	ADHD	T	25.4 (4.1)	–	20	–	Modified 1-back task	ERP	P1, N170, P2
Liu et al., 2016 (Study 1)	ADHD	D	24.2 (4.1);	18 to 35	136	Non-ADHD (41)	Delayed match-to-sample task	oscillations	alpha
Massand et al., 2013	ASD	D	25.7 (4.8)	–	22	Non-ASD (14)	Recognition memory test	ERP	Old-new ERP
Kulakova and Nieuwland, 2016	ASD	T	22.0 (4.0)	–	30	–	Comprehension task	ERP	N400
Gold et al., 2010	ASD	D	21.9 (3.0)	18 to 30	16	Non-ASD (16)	Semantic integration task	ERP	N400
Jochaut et al., 2015	ASD	D	20.7 (6.8)	15 to 40	13	Non-ASD (13)	Speech processing paradigm	oscillations	gamma, theta

*Note.* EEG = electroencephalography; ADHD = attention deficit/hyperactivity disorder; ASD = autism spectrum disorder; D = diagnosis; T = trait; ERP = event related potentials.

#### 3.7.1 ADHD

##### 3.7.1.1 ERP

Two studies examined WM, which is the ability to temporarily hold and manipulate information in mind. In a delayed match-to-sample task, participants were asked to judge whether target stimuli (abstract figures) were presented in previous trials (Kim et al., 2014). In addition to lower accuracy, ADHD at ages 19–35 was associated with typical P1 but reduced P3b amplitude in parietal-occipital sites during stimulus encoding, suggesting reduced allocation of attentional resources to encoding of information in WM. Another study examined the relationship between ADHD symptoms at age 25 and neural correlates of distractor processing using a modified 1-back task (with faces) (Biehl et al., 2013). In this task, participants were instructed to judge if a given target stimulus was also presented in the immediately preceding trial. The critical finding was that N170 amplitude was more pronounced for task-relevant stimuli compared to high-distracting task-irrelevant stimuli, with reduced N170 enhancement linked to more false alarms as well as more hyperactivity and impulsivity. Thus, reduced enhancement in N170 amplitude may reflect less successful WM maintenance also linked to the presence of ADHD-related traits.

##### 3.7.1.2 EEG oscillations

A study analysed event-related oscillatory activity in the maintenance phase of the delayed match-to-sample task described previously to investigate WM (Kim et al., 2014), that is, the time window 500–2000 ms after the onset of the to-be-remembered stimuli (Liu et al., 2016). ADHD at age 24 was associated with lower induced alpha power indexing reduced WM maintenance. These findings were interpreted as reduced efficiency of maintaining representation within WM during the retention period, as alpha is thought to play a role in inhibiting task-irrelevant information (Klimesch et al., 2007) (Table 1B).

#### 3.7.2 ASD

##### 3.7.2.1 ERP

One study investigated long-term memory of visually-presented words, and found that despite equivalent memory performance, ASD at 25 was associated with differential topographical neural activity patterns during recognition memory of old versus new items: in the parietal instead of anterior regions within 300–500 ms post-stimulus, and attenuated at the right frontal regions within 800–1500 ms post-stimulus (Massand et al., 2013). Such a pattern suggests either different neural basis for declarative memory in ASD, or the use of different retrieval strategies.

Another aspect investigated in ASD is language processing, using paradigms eliciting N400 – a putative marker of effort for processing and integrating meaning (Kutas and Federmeier, 2011) (Table 1A). For example, individuals with high ASD traits at age 22 showed reduced N400 amplitude to sentences describing counterfactual pretences, that is, alternative worlds known to be false (e.g., ‘if words were made out of sugar’). Such findings could potentially explain difficulties with pragmatic skills in ASD, referring to the ability to interpret communication of others (Kulakova and Nieuwland, 2016). Further, ASD at age 21 was associated with more negative N400 amplitudes to both novel and conventional metaphors compared to non-ASD controls (Gold et al., 2010), suggesting that metaphoric understanding poses difficulties on the semantic integration process in ASD.

##### 3.7.2.2 EEG oscillations

A study investigated gamma and theta oscillations during speech processing in ASD at age 20 (Jochaut et al., 2015). While non-ASD controls showed downregulation of gamma by theta in response to speech, the reverse pattern was observed for ASD. This atypical interaction of theta and gamma also correlated with the severity of ASD symptoms, which suggests that the lack of coordination between these two oscillations account for ASD-related impairments in speech processing.

## 4 Discussion

We set to conduct a systematic review of EEG-imaging studies (ERP and EEG oscillations) with ADHD and/or ASD. The EEG literature in these disorders is vast and has rapidly expanded since the early 2000’s, as revealed by the number of studies we have found despite restricting the sample to young adults. The studies were highly variable: the majority used varying participant samples, diagnostic procedures, and EEG analytical approaches (Table 1A, Table 1B). We were, however, able to group them in broad categories reflecting key neurocognitive domains (Tables 2A–2G), mostly reflecting putative core 'deficits' associated with each of the disorders. Critically, when the same neurocognitive domain has been considered in both disorders, rarely has the same paradigm been used, or often studies using similar paradigms would use distinct task parameters (e.g., stimuli type, modality, and timing).

What is most important now, we believe, is the need to consolidate this ever-evolving but disparate literature, and call for the fields of ADHD, ASD and EEG to come together with the common goal of understanding the overlap and distinction between the two disorders. Below we provide a synthesis of the candidate disorder-overlapping and disorder-specific neurophysiological markers of ADHD and ASD, as informed by the most up-to-date and comprehensive literature on EEG-imaging in young adult samples.

### 4.1 Summary of key findings

We intend to draw broad functional patterns across neurocognitive domains and paradigms in this general discussion. An overview of the putative cognitive-neurophysiological basis of ADHD and ASD, as well as their overlap and distinction, is presented in Table 3.

**Table 3:** Some key ERP/EEG findings to inform research into the neural basis of ADHD-ASD overlap and distinction.

Neurocognitive domain	Example of subprocesses investigated	Example of relevant ERP/EEG features	ADHD	ASD
Attentional processing	Cue processing	P3b, delta	x	
	Response preparation	CNV	x	
	Novelty processing	P3a		x
	Sustained attention	VLF	x	
	Perceptual binding	gamma		x

Inhibitory control		N2, LRP	x	

Performance monitoring	Conflict monitoring	N2, theta	x	
	Error processing	ERN, Pe	x	x
	Predictions	CNV, mu		x

Face processing	Structural encoding	N170, P1	x	x

Imitation and empathy	Pain perception	N2, P2, LPP		x
	Action observation	mu		x

Sensory processing	Visual processing	P1, theta, alpha	x	x
	Auditory processing	N1, theta, alpha	x	x
	Context modulation	gamma		x

Memory and language	Working memory	alpha; P3b	x	
	Semantic processing	N400		x
	Speech processing	gamma, theta		x

*Note*. Cells marked with 'x' indicate evidence for atypical profile for a given disorder within an investigated subprocess; empty cells suggest that a subprocess has not been investigated in a given disorder (using EEG-imaging in young adults); this table points to disorder-specific and disorder-overlapping profiles, but direct cross-disorder comparisons using the same paradigm are required; ADHD = attention deficit hyperactivity disorder; ASD = autism spectrum disorder; EEG = electroencephalography; ERP = event-related potentials; CNV = contingent negative variation; VLF = very-low frequency oscillations; LRP = lateralised readiness potential; ERN = error-related negativity; Pe = error positivity; LPP = late positive potential.

Both ADHD and ASD have been linked to atypical allocation of attentional resources and atypical performance monitoring, but there are also atypical processing aspects that are disorder-specific. In ADHD, much atypical processing in the attentional domain relates to orienting to cues that signal response preparation, whereas in ASD it appears to be more specific to novelty, an effect that may be at least partially influenced by increased perceptual capacity (and increased maintenance of visual stimuli in WM). ADHD may relate to atypical monitoring of conflict and of rewarding feedback, whereas ASD may relate to atypical feedback processing involving socially-relevant stimuli. Additionally, ADHD has also been linked to diminished inhibitory control, which has not been directly studied in ASD during young adulthood.

Both ADHD and ASD have also been linked to atypical processing of faces, and atypical early sensory processing in both visual and auditory domains. However, disorder-specific profiles may qualify each disorder. In ASD, the evidence for atypical face processing is mixed, at least in young adulthood and may depend on the self-relevance quality of the stimuli, whereas in ADHD the atypical processing may be specific to anger emotions. For ASD, atypical sensory processing has been suggested by various studies to contribute to ‘later’ cognitive deficits such as face processing and attentional processing. Some domains have been studied exclusively in ASD, thus direct comparisons with ADHD are required, including evidence of atypical language/semantic processing and mixed findings on atypical imitation processing.

### 4.2 Methodological considerations

A critical gap in the literature is investigation across disorders. Some studies have compared either ADHD with other clinical groups (Iacono et al., 2002; Rommel et al., 2017), and ASD with other clinical groups (Magnée et al., 2009), thus informing to a degree the issue of specificity. However, not a single study directly compared ADHD with ASD in young adulthood using EEG-imaging. Individuals with both disorders have also not been included, which therefore limits our understanding of, for example, whether they represent a distinct clinical group or instead are better understood as the combination of both disorders in isolation (Tye et al., 2014a, 2014b, 2013). This issue is the most critical for future research aiming to establish the neural basis of ADHD and ASD, both in isolation and together.

The other major limitation of the literature is the overly large sampling interval used in some studies with young adult samples, despite the mean age falling within our specified cut-off (ages 16–26) (Tables 2A–2G). Some of the studies, particular in the ASD literature, included a broad age range (overlapping with children and late adult samples) even if the mean age fell within our pre-specified period. The brain undergoes changes across the lifespan even after childhood and adolescent (Arnett, 2007; Casey et al., 2008; Giedd, 2008; Rubia et al., 2000; Sowell et al., 2003; Taber-Thomas and Pérez-Edgar, 2014). A truly developmental perspective demands characterisation of cognitive and neural processes pertaining specifically to emerging adults as they transition (or not) into independence, ideally with an even more dense sampling within the 16–26 age range. We opted for a more liberal approach in this review because of the paucity of the relevant literature in young adults, and hope this can galvanise improved research design.

One important issue in relation to age is that the prefrontal cortex develops throughout adolescent until around the age of 25 (Casey et al., 2008), and thus the brain of young teenagers (e.g., ages 16–19) can be different from the brain of those at the later phase of young adulthood (e.g., age 24). Therefore, disorder-specific atypical profiles of neurocognitive processing may also change with age, for instance due to changes in the prefrontal cortical maturation. Relevant to this point, a recent meta-analysis (which included participants with ages 17–57) showed that reduced P3b amplitude in ADHD relative to non-ADHD participants is exacerbated with age (Szuromi et al., 2011). The P3b ERP component is thought to be subserved by a ventral attention network, which receives input signals from prefrontal cortices (Corbetta et al., 2008). Given reported delays in prefrontal cortical maturation in ADHD (Seidman et al., 2005), atypical prefrontal contributions to the P3 ERP may become more apparent at the later phase of young adulthood (e.g., after age 20) compared to an earlier adolescent period when prefrontal cortices are yet fully developed (Casey et al., 2008). More studies with each specific age within the young adulthood period (such as 16–26) would be important to track atypical patterns that emerge due to developmental trajectories in (frontal) brain maturation, in particular using tasks that probe for executive functions and/or decision-making processes, which have been associated with frontal brain regions (Alvarez and Emory, 2006; Volz et al., 2006). It also remains important to ascertain whether these patterns are specific for example to disorders such as ADHD, by including more cross-disorder comparisons (Iacono et al., 2002; Rommel et al., 2017), including with ASD.

A neglected issue is the role of the informant in biomarker research. This review revealed that, at least in ADHD, the reliability of neurophysiological indices across various cognitive domains, particularly those based on ERP components, will differ depending on whether group classifications are based on informants (e.g., parents) (Cheung et al., 2016) rather than self-reported symptoms (Du Rietz et al., 2016). This issue has not been addressed in ASD; even though group classification in some studies for both disorders are based on self-reported trait measures. We therefore believe that simultaneous consideration of both formal diagnosis and trait measures are likely to be most informative in future studies.

The various research considerations described above would require large samples for appropriate statistical power, a common problem in neuroimaging studies. The majority of studies in our review involved small samples, typically recruited via convenient sampling methods (e.g., via a clinical site or within an university, leading to samples that may not be representative of all young people with ADHD and/or ASD), with a few exceptions (e.g., Boecker et al., 2014; Cheung et al., 2016; Michelini et al., 2016). As young people underuse healthcare services and some could find a diagnosis stigmatising, research in this age group is also likely to benefit from a combination of clinical and population-based samples.

### 4.3 Future directions

#### 4.3.1 Task paradigms

The review points towards opportunities to consider the design of task paradigms, assessing various neurocognitive domains, which are likely to inform our understanding of the ADHD-ASD co-occurrence and its neural basis. These designs, for example, may include experimental manipulation of task parameters building on the existing literature. We will briefly go over some possibilities in relation to each domain.

For attentional processing, the most common paradigm used across ADHD and ASD is variations on the oddball paradigm. Thus, one may consider task parameters that could reveal disorder-specific atypical processing. For instance, ADHD-specific profiles may depend on stimuli modality (visual vs. auditory) and ASD-related atypical attentional processing may be modulated by task load (Karhson and Golob, 2016) and/or stimuli novelty (Sokhadze et al., 2009a). An established attentional paradigm is the ADHD literature is the cued CPT (e.g., McLoughlin et al., 2010), which include trial types that distinguish various aspects of attentional processing, including cue orienting (cue-trials), response execution (go-trials) and response inhibition (no-go trials), and thus could help isolate potential atypical attentional processing that are ADHD-specific from ASD-specific. The consideration of trial conditions with inhibitory versus response demands can help further establish if indeed ADHD is associated with specific difficulties with inhibitory control, or instead with broader regulation problems regardless of the attentional demands (Fisher et al., 2011). Further, cross-disorder studies on performance monitoring may consider simultaneous assessments of various facets of monitoring within the same task, including conflict (Michelini et al., 2016), errors (Groom et al., 2010) and feedback (Broyd et al., 2012), as well as the monitoring context (e.g., social vs. non-social) (Carter Leno et al., 2016), as this combination could elucidate conditions under which ADHD and ASD are distinguished at the neural level.

Task paradigms that are broadly relevant to socioemotional processing might also combine parameters that have been considered separately in the ADHD and ASD literature. Face processing tasks could, for example, consider a range of positive and negative emotions, so to establish if processing of specific emotions are disorder-specific (e.g., Raz and Dan, 2015a, 2015b). Additional parameters to consider are passive viewing versus active recognition and faces versus non-faces, given the inconsistency, even in the ASD literature, in relation to atypical face processing (e.g., Faja et al., 2016; O’Connor et al., 2005; Stavropoulos et al., 2016; Webb et al., 2012). Similarly, paradigms that aim to assess imitation/empathy may include conditions that allow for more precise functional interpretation, for example by considering both non-emotional (e.g., action observations; Bernier et al., 2007) and emotional conditions (e.g., pain observation; Fan et al., 2014), as well as measurements of different facets of empathy, such as emotional identification versus affect sharing (Coll et al., 2017).

Finally, atypical sensory processing across auditory and visual domains have been reported in both ADHD and ASD. One issue is the heterogeneity in stimuli employed, spanning colour (Kim et al., 2015), orientation (Snijders et al., 2013), brightness (Gonen-Yaacovi et al., 2016), pitch (Alain et al., 2001), motion (Yamasaki et al., 2011), cross-modality integration (Magnee et al., 2008), and so forth. Researchers may wish to include a variety of stimuli in future cross-disorder studies. An important facet of stimuli processing – which could be relevant regardless of stimuli type – is the higher degree of moment-to-moment processing of the stimuli, also known as inter-trial variability. This review has identified evidence of increased inter-trial variability in EEG-based markers of stimuli processing in ADHD in young adults (Gonen-Yaacovi et al., 2016). Although not identified in our review, others have proposed that similar processes are involved also in ASD (David et al., 2016), and thus an important future direction is the comparison of neural variability across disorders.

An advantage of including additional task conditions is the improvement in functional interpretation of dimensions of neurocognitive processing to each disorder. However, we acknowledge that it might not always be practical/viable to include such conditions, for example, because of the additional burden to the participant during data collection (e.g., lengthening the duration of a task). Therefore, task parameters should be considered on a case-by-case basis depending on the main research aim, the resources available to the study, and the nature of the participant sample. One could consider smaller-scale studies to establish functional interpretation for paradigms for which findings are more controversial, in combination with larger-scale studies using more established paradigms for whch findings have more broadly-accepted functional interpretations.

#### 4.3.2 Developmental psychopathology

We also suggest further research considerations to better understand the neural basis of ADHD and ASD (and their co-occurrence) within a developmental psychopathology framework. For example, genetically-informed studies can be used to clarify the etiology of the overlap and distinction between ADHD and ASD, by helping tease apart the genetic and environmental contributions to these disorders and their co-occurrence in young adulthood. Family studies or twin studies, in combination with EEG-imaging, could be used to estimate whether the neurocognitive profiles associated with ADHD and ASD stem from common or distinct aetiologies. Such study designs could shed lights on the neurocognitive mechanisms underlying the shared genetic influences between the disorders (Rommelse et al., 2010).

Additional longitudinal research, both within and across ADHD and ASD, is also required to track changes in neurophysiological processes from childhood into adulthood. Longitudinal studies are particularly relevant in light of persistence of symptoms despite a drop in diagnosis (e.g., Asherson, 2016), potential development of compensatory strategies (Livingston and Happé, 2017), and the recent proposed controversial distinction between childhood-onset versus adulthood-onset ADHD (e.g., Moffitt et al., 2015). While this review has identified some longitudinal studies (Boecker et al., 2014; Cheung et al., 2017, 2016; Gilmore et al., 2011; Iacono et al., 2002; James et al., 2017; Michelini et al., 2016), none of these reported more than one data point with EEG-imaging data, or considered ASD. Thus, conclusions about potential changes (or stability) in neurocognitive processing, across development in relation to each disorder, are currently limited.

Finally, given the higher incidence of mental health risk in ADHD and ASD (Anckarsäter et al., 2006; Davidson, 2008; El Achkar and Spence, 2015; Levy and Perry, 2011; Murray, 2010; van der Meer et al., 2012), future research is likely to profit from routine assessments of co-occurring mental health difficulties as well as bridging with the established EEG-imaging literature in, for example, affective disorders research. Assessments of mental health would help delineate atypical neurocognitive processing that is specific to ADHD and/or ASD, rather than explained by, for instance, co-occurring mood disorders, which have also been linked to atypical neurocognitive processing in similar domains considered in this review (e.g., Meyer, 2017; Olbrich and Arns, 2013). It would also help expand on our understanding of the relationship between these disorders and functional aspects of wellbeing beyond core symptoms of the psychopathology that, for example, may be informative in clinical contexts (Thapar et al., 2017).

#### 4.3.3 Recent advances in EEG-imaging

Recent hardware developments in mobile EEG have led to new portable, light-weight and increasingly affordable EEG systems. Mobile EEG technologies might constitute an ideal tool to ease data collection from large number of participants within a relatively short timeframe, and within powerful developmentally-informative research design (McLoughlin et al., 2014a), such as a longitudinal twin study. Mobile EEG can also be used in flexible locations, and thus more inclusive of participants with ADHD or ASD whom may prefer to not travel to a research centre. Such ease of data collection could help encourage the field to move away from small sample sizes, and to initiate cross-site collaborative efforts with large-scale EEG studies.

EEG-imaging research is currently exploiting recent advances in signal processing, which are likely provide additional information compared to traditional analytical approaches. Such ‘microscopic’ examination, exploiting all available EEG data, provides novel opportunities to better link the underlying neurophysiology of these disorders with their cognitive and behavioural profiles, as well as with their overlap and distinction. Among these approaches, ICA can be used to separate neural from non-neural activity, increasing the signal-to-noise ratio of the neural components (Makeig et al., 2004), thus aiding identification of the neural sources. This approach also permits robust single trial analyses to consider neural variability – a key proposed cognitive feature of ADHD (Gonen-Yaacovi et al., 2016). A source-based approach has shown to be more informative of neurophysiological processes underlying genetic risk of ADHD than scalp-based data (e.g., McLoughlin et al., 2014b), but is yet to be leveraged for ASD research. Other approaches included in this review were the use of time-frequency decomposition (Gilmore et al., 2011), measures of neural connectivity (Jaime et al., 2016; Nowicka et al., 2016), and proposed solutions to source localisation (Broyd et al., 2011).

### 4.4 Limitations of the review

We have focused exclusively on EEG-imaging, but other imaging modalities such as fMRI or MEG, which are typically thought as having better spatial resolution, can also contribute to research into the neural basis of the ADHD-ASD overlap and distinction (Cortese et al., 2012; Kikuchi et al., 2016; Paloyelis et al., 2007; Philip et al., 2012). The use of EEG-imaging and its recent mobile developments in large samples can serve as a foundation for more targeted analyses with other high-cost and less accessible methods (e.g., Bridwell et al., 2013).

Our review consists of a qualitative rather than a quantitative synthesis of the available evidence. While we have identified a relatively high number of studies in young adult samples (75 articles), these were spread across neurocognitive domains and have employed heterogeneous cognitive paradigms – precluding pooling of the data across studies at this stage, for instance, via meta-analyses. Here, we highlight the candidate neurocognitive processes and markers leveraging EEG-imaging, alongside established and novel cognitive paradigms, which could provide the foundation for the next phase of research in ADHD-ASD overlap. We also seek to galvanise joint collaborative efforts, such as data-sharing, so that a more formal quantitative reviews could be conducted in the near future. As the literature in this age group accumulates, further reviews could focus on specific age ranges and specific neurocognitive processes/tasks within this developmental period.

Despite reviewing a relatively large number of studies, we may have missed out on important studies that are nevertheless relevant to our aim of understanding the overlap between ADHD and ASD. For example, intra-participant variability in sensory processing (as assessed by ITC) was discussed in the context of ADHD (Section 3.6.1.2) but not in ASD, even though recent studies have shown that it could also be a feature of ASD (for a recent review see David et al., 2016). These studies would have been missed due to our exclusion criteria, which did not permit studies using instead MEG (Edgar et al., 2015; Gandal et al., 2010; Rojas et al., 2008; Sun et al., 2012) or those with samples which were older (Buard et al., 2013) or younger (Milne, 2011) than our pre-specified age range. We acknowledge that the study of ADHD and ASD (and their neural basis) in young adulthood would also benefit from considering task paradigms/analytical approaches not yet used with EEG-imaging and/or in this age group.

## 5 Conclusions

EEG-imaging studies in ADHD and ASD are rapidly accumulating, reflecting the field’s enthusiasm to leverage EEG methods, and the potential of this research to inform the neural basis of the ADHD-ASD overlap and distinction. Despite the lack of studies directly comparing both disorders or including dual diagnosis, some consistent candidate disorder-overlapping and disorder-specific biomarkers have emerged. Moving forward, research into overlap and distinction that harnesses EEG-imaging should focus on cross-disorder comparisons using the similar paradigms, better powered studies and larger samples, more restricted age ranges, well-characterised longitudinal cohorts, and further applications of time-frequency and source-based EEG approaches.
